# Dependence of Intracellular and Exosomal microRNAs on Viral *E6/E7* Oncogene Expression in HPV-positive Tumor Cells

**DOI:** 10.1371/journal.ppat.1004712

**Published:** 2015-03-11

**Authors:** Anja Honegger, Daniela Schilling, Sandra Bastian, Jasmin Sponagel, Vladimir Kuryshev, Holger Sültmann, Martin Scheffner, Karin Hoppe-Seyler, Felix Hoppe-Seyler

**Affiliations:** 1 Molecular Therapy of Virus-Associated Cancers (F065), German Cancer Research Center (DKFZ), Heidelberg, Germany; 2 Cancer Genome Research (B063), German Cancer Research Center (DKFZ) and German Cancer Consortium (DKTK), Heidelberg, Germany; 3 Department of Biology, University of Konstanz, Konstanz, Germany; Cincinnati Children’s Hospital, UNITED STATES

## Abstract

Specific types of human papillomaviruses (HPVs) cause cervical cancer. Cervical cancers exhibit aberrant cellular microRNA (miRNA) expression patterns. By genome-wide analyses, we investigate whether the intracellular and exosomal miRNA compositions of HPV-positive cancer cells are dependent on endogenous *E6/E7* oncogene expression. Deep sequencing studies combined with qRT-PCR analyses show that *E6/E7* silencing significantly affects ten of the 52 most abundant intracellular miRNAs in HPV18-positive HeLa cells, downregulating miR-17-5p, miR-186-5p, miR-378a-3p, miR-378f, miR-629-5p and miR-7-5p, and upregulating miR-143-3p, miR-23a-3p, miR-23b-3p and miR-27b-3p. The effects of *E6/E7* silencing on miRNA levels are mainly not dependent on p53 and similarly observed in HPV16-positive SiHa cells. The *E6/E7*-regulated miRNAs are enriched for species involved in the control of cell proliferation, senescence and apoptosis, suggesting that they contribute to the growth of HPV-positive cancer cells. Consistently, we show that sustained *E6/E7* expression is required to maintain the intracellular levels of members of the miR-17~92 cluster, which reduce expression of the anti-proliferative *p21* gene in HPV-positive cancer cells. In exosomes secreted by HeLa cells, a distinct seven-miRNA-signature was identified among the most abundant miRNAs, with significant downregulation of let-7d-5p, miR-20a-5p, miR-378a-3p, miR-423-3p, miR-7-5p, miR-92a-3p and upregulation of miR-21-5p, upon *E6/E7* silencing. Several of the *E6/E7*-dependent exosomal miRNAs have also been linked to the control of cell proliferation and apoptosis. This study represents the first global analysis of intracellular and exosomal miRNAs and shows that viral oncogene expression affects the abundance of multiple miRNAs likely contributing to the *E6/E7*-dependent growth of HPV-positive cancer cells.

## Author Summary

Oncogenic human papillomaviruses (HPVs) are major human carcinogens of broad biomedical importance. The growth of HPV-positive cervical cancer cells is critically dependent on sustained *E6/E7* oncogene expression from endogenous viral DNA sequences. We here addressed the question of whether this process is linked to specific, *E6/E7*-dependent alterations of the cellular micro(mi)RNA network. By comprehensive deep sequencing analyses we show that endogenous *E6/E7* expression significantly affects the concentrations of abundant intracellular miRNAs in HPV-positive cervical cancer cells, which are linked to the control of cell proliferation, senescence and apoptosis. These include members of the miR-17~92 cluster, which are expressed at increased levels by sustained *E6/E7* expression and repress the anti-proliferative *p21* gene in HPV-positive cancer cells. Moreover, we identified an *E6/E7*-dependent seven-miRNA-signature in exosomes secreted from HPV-positive cancer cells. These small vesicles are involved in intercellular communication and may serve as novel diagnostic markers. Taken together, our results show that continuous *E6/E7* expression in HPV-positive cancer cells is linked to significant alterations in the amounts of intracellular and exosomal miRNAs with growth-promoting, anti-senescent and anti-apoptotic potential.

## Introduction

Oncogenic human papillomaviruses (HPVs), such as HPV16 and HPV18, cause cervical cancer. Infections with oncogenic HPV types are moreover closely linked to the development of additional human malignancies in the oropharynx and anogenital region [1]. The viral E6 and E7 oncoproteins are crucial both for the HPV-associated induction of transformation as well as for the maintenance of the tumorigenic phenotype of HPV-positive cervical cancer cells [2,3]. For example, E6 induces the proteolytic degradation of the p53 tumor suppressor protein [4] and stimulates telomerase activity [5], whereas E7 interferes with the activity of the retinoblastoma tumor suppressor protein, pRb, and other pocket proteins [6]. As a consequence, E6 and E7 deregulate intracellular pathways involved in the control of cellular proliferation, senescence, apoptosis, and genetic stability.

Importantly, at least some of these pathways are not irreversibly impaired by HPVs. Rather, inhibition of viral *E6/E7* activities in HPV-positive cancer cells leads to the reactivation of dormant tumor suppressor pathways. For instance, several studies indicate that inhibition of E6 primarily results in apoptosis [7–11], whereas combined inhibition of E6/E7 leads to growth arrest and cellular senescence [12–14]. The reversibility of the malignant phenotype of HPV-positive tumor cells is not only phenomenologically interesting but may also form a rational basis for therapeutic interference. This could, in principle, be achieved by blocking the *E6/E7* oncogenes or, alternatively, by correcting downstream cellular pathways that are deregulated by the viral oncogenes. Therefore, it is important to uncover crucial cellular targets that are affected by viral *E6/E7* oncogene expression and that support the growth of HPV-positive cancer cells.

Micro(mi)RNAs are short (21–23 nt), non-coding, highly-conserved RNAs that post-transcriptionally regulate gene expression [15]. For several tumor entities, it has been shown that the deregulation of the cellular miRNA network plays a critical role for cancer development and maintenance [16,17]. The oncogenicity of miRNAs has been particularly well demonstrated for members of the miR-17~92 cluster (also called “oncomir-1”; coding for miR-17, miR-20a, miR-18a, miR-19a, miR-19b and miR-92a) and of its paralog cluster miR-106b~25 (coding for miR-106b, miR-93 and miR-25) [18]. Potential cellular target genes for members of the two miRNA clusters include *p21*, which codes for a cyclin-dependent kinase inhibitor that plays a central role for growth control and induction of the senescence pathway in many cells [19,20].

In contrast to other tumor viruses, such as Epstein-Barr virus (EBV) or Kaposi’s sarcoma-associated herpesvirus (KSHV), oncogenic HPV types presumably do not encode own viral miRNAs [21,22]. However, global miRNA analyses indicate an upregulation of oncogenic miRNAs and a decrease of tumor-suppressive miRNAs in cervical cancer biopsies [23–38] and in cervical cancer cell lines [39–41], in comparison to normal cervical tissue. A contribution of HPVs to the deregulation of miRNA expression in cervical cancer was proposed, mainly based on comparisons between HPV-positive and -negative cell lines [41], and on cell culture models upon introduction of HPV genomes [22,42,43]. More direct evidence that the HPV oncogenes have the potential to influence the cellular miRNA composition was provided by experiments that involved ectopic expression of the viral E6 and/or E7 genes in keratinocytes and subsequent global miRNA analysis [44] or investigation of selected miRNAs [45,46]. However, the miRNA species identified by these different experimental approaches vary substantially (see Discussion). Most importantly, the crucial question whether the E6/E7-dependent maintenance of the growth of HPV-positive cancer cells is linked to specific alterations of the global miRNA network has not been addressed. To resolve this issue, we here performed a comprehensive analysis of the intracellular miRNA composition in HPV-positive cancer cells, upon silencing of endogenous *E6/E7* oncogene expression.

An interesting miRNA pool that recently gained interest in cancer research is the miRNA content of exosomes. Exosomes are small extracellular vesicles (50–100 nm in diameter) of endosomal origin that are secreted by a variety of cells, including tumor cells [47]. Exosomes may play an important role for the intercellular communication of tumor cells since they can accelerate cancer growth and invasiveness by horizontally transferring proteins, mRNAs, and non-coding RNAs from tumor cells into recipient cells [48–50]. In the case of miRNAs, several studies showed specific target gene repression in recipient cells upon intercellular transfer of miRNAs via exosomes [51–55]. Also other human tumor viruses, EBV [51,56,57] and possibly KSHV [58], may utilize exosomes to modulate the tumor microenvironment by transporting viral proteins and virus-encoded miRNAs. Due to the facts that exosomes can be isolated from different body fluids (e.g. serum, saliva, urine) and that their content allows conclusions about their cell of origin, exosomes are also intensively investigated as a source of novel biomarkers [59–61].

The above considerations raise two important issues concerning the interplay between HPVs and the miRNA network in cervical cancer cells. First, is the intracellular miRNA pool of HPV-positive tumor cells dependent on the sustained expression of the viral *E6/E7* oncogenes? Second, is the miRNA composition of exosomes that are secreted by HPV-positive cancer cells affected by the HPV oncogenes? To answer these questions, we performed a comprehensive deep sequencing study in order to identify the influence of the endogenous *E6/E7* oncogene expression on the global miRNA composition of HPV-positive cervical cancer cells, both at the intracellular and at the exosomal level.

## Results

### Influence of endogenous *E6/E7* expression on the intracellular miRNA content of cervical cancer cells

**Fig 1:**
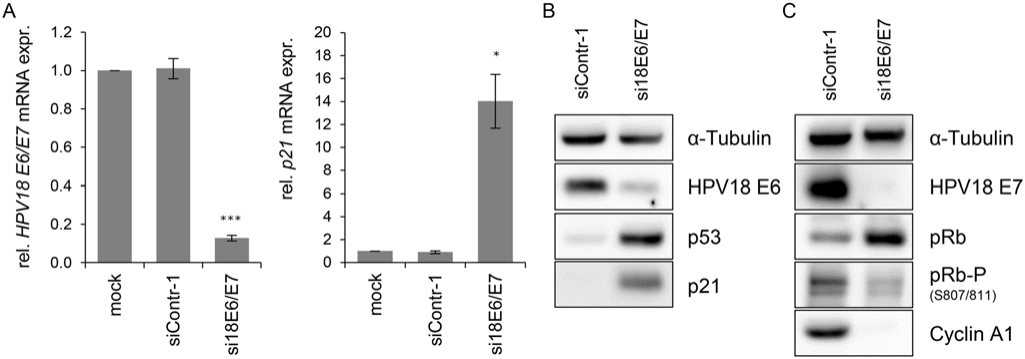
Silencing of HPV18 *E6/E7* expression by RNA interference. **(A)** qRT-PCR analysis of HPV18 *E6/E7* (left panel) and *p21* (right panel) mRNA expression, 72 h after transfection of HeLa cells with si18E6/E7, control siRNA siContr-1, or upon mock treatment. mRNA levels were normalized to *ACTB* and calculated relative to the mock control. Data represent mean ± SEM (n = 4). Asterisks above columns indicate statistically significant differences from siContr-1-treated cells (p ≤ 0.05 (*), p ≤ 0.001 (***)). **(B)** Immunoblot analysis of HPV18 E6, p53, and p21 protein levels, 72 h after transfection of HeLa cells with si18E6/E7 or siContr-1. α-Tubulin: loading control. **(C)** Immunoblot analysis of HPV18 E7, total pRb (pRb), phosphorylated pRb (pRb-P), and Cyclin A1 protein levels, 72 h after transfection of HeLa cells with si18E6/E7 or siContr-1. α-Tubulin: loading control.

In order to investigate the influence of the HPV oncogenes on the intracellular miRNA composition of HPV-positive cancer cells, endogenous HPV18 *E6/E7* expression in HeLa cervical carcinoma cells was blocked by RNA interference (RNAi) for subsequent deep sequencing analyses. Treatment with *E6/E7*-targeting siRNAs (si18E6/E7) led to efficient downregulation of *E6/E7* mRNA levels as shown by qRT-PCR analyses, using primers recognizing all three transcript classes [8] coding for HPV18 E6 and E7 (Fig. 1A, left panel). A substantial reduction of the HPV18 E6 and E7 protein levels 72 h after transfection with si18E6/E7 was observed (Fig. 1B/C). This was linked to increased p53 protein levels (Fig. 1B), as expected from the ability of E6 to induce the degradation of p53 [4], and increased *p21* mRNA and protein levels (Fig. 1A/B), representing a downstream transcriptional target gene for p53 [62]. Further, *E6/E7* silencing was associated with an increase of total pRb protein levels, consistent with the ability of E7 to induce pRb degradation [6], as well as with decreased amounts of phosphorylated pRb and of Cyclin A1, indicating reactivation of the pRb cascade (Fig. 1C).

cDNA libraries were generated from RNA samples isolated from cells in which the viral oncogene expression was silenced (si18E6/E7) or which underwent control treatment (siContr-1). In order to capture a broad spectrum of *E6/E7*-modulated miRNAs—unbiased by a pre-selection—analyses of total miRNA contents were accomplished by small RNA deep sequencing using the Illumina platform. Sequencing of the libraries resulted in raw read counts that were pre-processed to remove adapter sequences and filtered to exclude low quality reads (S1 Table).

**Fig 2:**
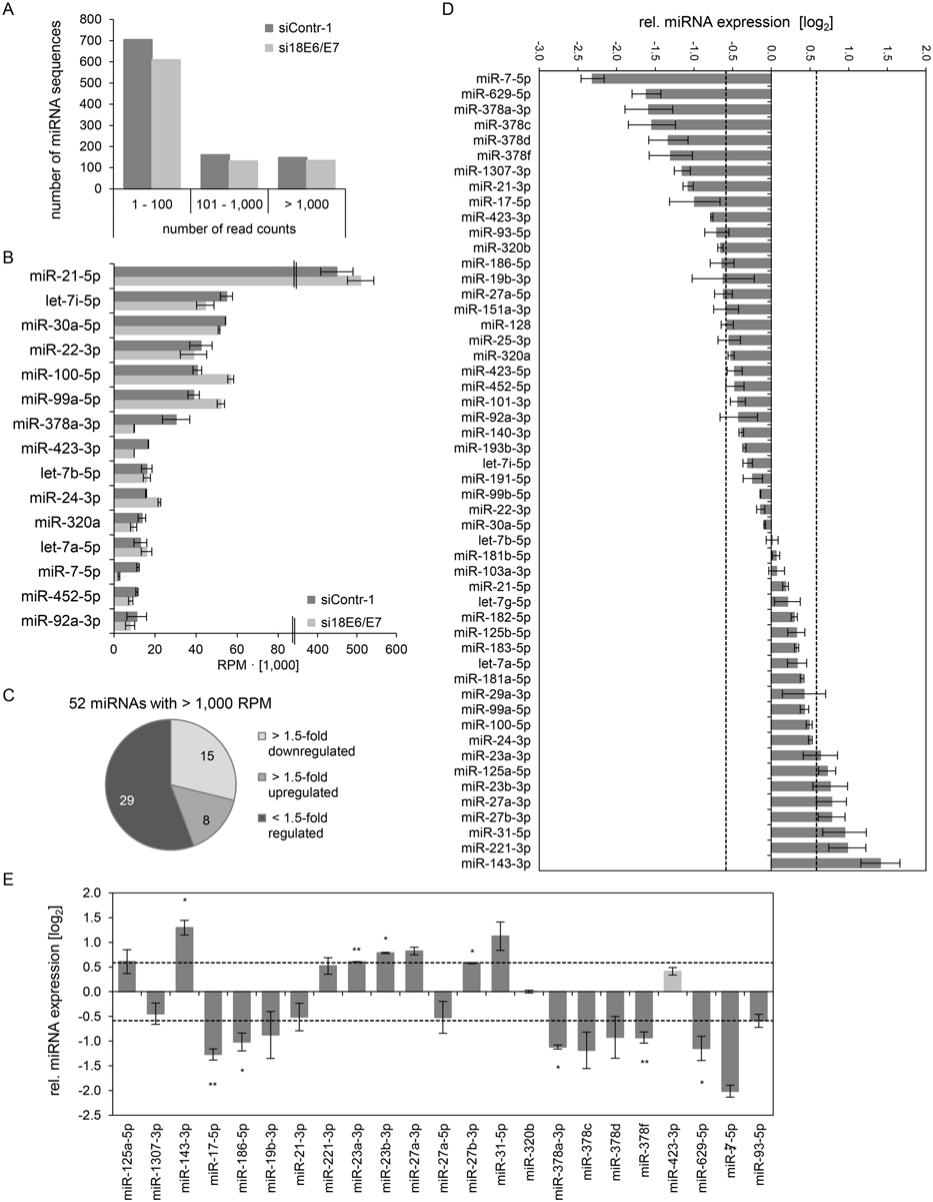
Inhibition of endogenous HPV18 *E6/E7* expression: Effects on the intracellular miRNA composition of cervical cancer cells. Small RNA deep sequencing (A—D) and qRT-PCR analyses (E) of cellular miRNAs, 72 h after transfection of HeLa cells with si18E6/E7 or control siRNA siContr-1. **(A)** Mean read count distribution of mature miRNA sequences in si18E6/E7- and siContr-1-transfected cells (n = 2). Only miRNAs with a mean read count > 1 were considered. **(B)** The 15 most frequently sequenced cellular miRNAs. Selection based on siContr-1 samples, respective values for the si18E6/E7-treatment are indicated. Data represent mean ± SEM (n = 2). Interrupted x-Axis. **(C)** Overview on differentially affected (> 1.5-fold) cellular miRNAs, determined by small RNA deep sequencing. RPM values of si18E6/E7-treated samples were calculated relative to the control treatment (siContr-1). Only miRNAs with > 1,000 RPM in each sample were considered (n = 2). **(D)** Relative quantification of miRNAs in si18E6/E7- versus siContr-1-treated cells as assessed by small RNA deep sequencing (log_2_ display). Dashed lines: 1.5-fold up- or downregulation (log_2_(1.5) = 0.585). Only miRNAs with > 1,000 RPM in each sample were considered. Data represent mean ± SEM (n = 2). **(E)** qRT-PCR analyses of *E6/E7*-dependent cellular miRNAs identified by small RNA deep sequencing. Cellular miRNA levels were normalized to snRNA *RNU6–2* and calculated relative to siContr-1 (log_2_ display). Dashed lines: 1.5-fold up- or downregulation (log_2_(1.5) = 0.585). The column color shows regulation in the same (dark grey) or opposite (light grey) direction compared to the small RNA deep sequencing data of the individual miRNAs. Data represent mean ± SEM (n = 2 or 3). Asterisks indicate statistically significant differences (p ≤ 0.05 (*), p ≤ 0.01 (**) and p ≤ 0.001 (***)).

Mean read counts of cellular sequences mapping to known human miRNAs ranged from 1 to 1,170,975 (S1 Dataset, Fig. 2A for a read count distribution of cellular miRNAs). For cross-library comparison, the read count of a given miRNA was normalized to the total number of uniquely mapped miRNA reads per library and expressed as reads per million (RPM) mapped reads. RPM values of the 15 most frequently sequenced cellular miRNAs are displayed in Fig. 2B.

For stoichiometric reasons, it is assumed that mainly miRNAs with a high intracellular abundance can lead to the repression of their target genes, whereas low-abundant miRNAs, identified in deep sequencing studies with < 100 RPM, are frequently not functional [63]. Therefore, an arbitrary threshold of 1,000 RPM for an individual miRNA in each sample was set and, consequently, 52 cellular miRNAs were further analyzed. Deregulation was defined as a > 1.5-fold change between si18E6/E7- and siContr-1-treated samples. An overview on the number of deregulated cellular miRNAs is given in Fig. 2C, showing that endogenous *E6/E7* silencing affected 23 of the 52 most abundant miRNAs in HeLa cells (15 down- and 8 upregulated). The relative expression upon *E6/E7* silencing of each of the 52 miRNAs is displayed in Fig. 2D. The RPM_mean_ values and fold changes of the 23 *E6/E7*-regulated miRNAs are indicated in S2 Table.The 23 miRNAs comprise several family members of the miR-378 family (miR-378a-3p, miR-378c, miR-378d, miR-378f), as well as members of the miR-17~92 and miR-106b~25 clusters (miR-17–5p, miR-19b-3p, miR-93–5p). Moreover, two seed families, the miR-23 family (miR-23a-3p, miR-23b-3p) and miR-27 family (miR-27a-3p, miR-27b-3p), were upregulated.

The *E6/E7*-mediated modulation of cellular miRNAs was subsequently validated by qRT-PCR analyses. For normalization, small nuclear RNA (snRNA) *RNU6–2* was employed. Ct-values for *RNU6–2* were consistent for si18E6/E7- or siContr-1-treated samples and across experimental conditions. Of the 23 abundant cellular miRNAs that were found to be affected in deep sequencing analyses, deregulation (up or down) upon silencing of endogenous *E6/E7* expression was confirmed for 21 miRNAs (91%) by qRT-PCR (dark grey columns in Fig. 2E), 17 of which exhibited a fold change of > 1.5 (Fig. 2E and S2 Table). This shows high agreement between the two methods. Statistical significance of the qRT-PCR data was obtained for ten of these 17 miRNAs: downregulation of miR-17–5p, miR-186–5p, miR-378a-3p, miR-378f, miR-629–5p and miR-7–5p and upregulation of miR-143–3p, miR-23a-3p, miR-23b-3p and miR-27b-3p, upon *E6/E7* silencing (Fig. 2E and indicated in bold in S2 Table). In summary, the combination of small RNA deep sequencing and qRT-PCR analyses identified ten abundant cellular miRNAs that are significantly affected in HeLa cells upon silencing of endogenous *E6/E7* oncogene expression.

**Fig 3:**
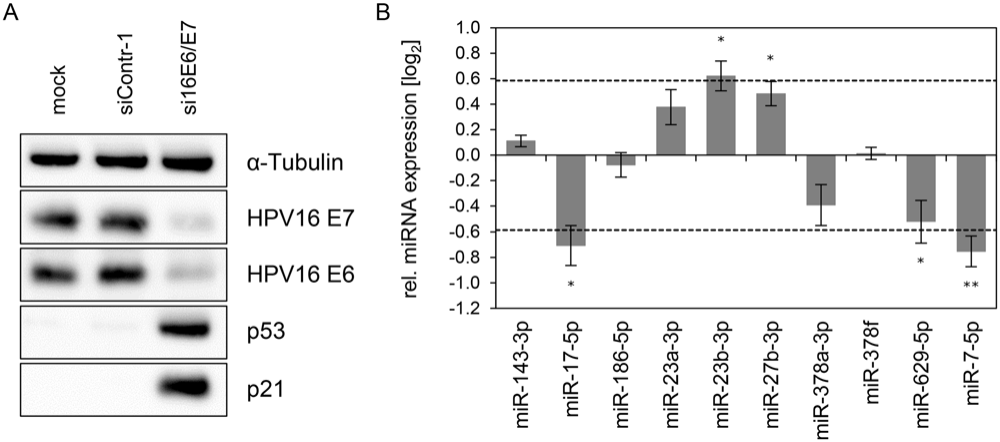
Inhibition of endogenous HPV16 *E6/E7* expression: Effects on selected intracellular miRNAs. **(A)** Immunoblot analysis of HPV16 E7, HPV16 E6, p53 and p21 protein levels, 72 h after transfection of SiHa cells with si16E6/E7 or control siRNA (siContr-1), or upon mock treatment. α-Tubulin: loading control. **(B)** qRT-PCR analyses of ten selected cellular miRNAs, 72 h after transfection of SiHa cells with si16E6/E7 or siContr-1. Cellular miRNA levels were normalized to the snRNA *RNU6–2* and calculated relative to siContr-1 (log_2_ display). Dashed lines: 1.5-fold up- or downregulation (log_2_(1.5) = 0.585). Data represent mean ± SEM (n = 3). Asterisks indicate statistically significant differences (p ≤ 0.05 (*) and p ≤ 0.01 (**)).

Next, we analyzed the expression of these ten miRNAs upon repressing endogenous *E6/E7* expression in SiHa cervical cancer cells (Fig. 3). In contrast to HPV18-positive HeLa cells, which are derived from an adenocarcinoma of the cervix, SiHa cells express HPV16 *E6/E7* and originate from a cervical squamous cell carcinoma. As expected, silencing of endogenous HPV16 *E6/E7* expression by RNAi in SiHa cells was linked to reduced E6/E7 protein expression and reconstitution of the p53 pathway (Fig. 3A). Notably, both cell lines reveal a substantial overlap in the regulation patterns of the ten selected miRNAs, upon endogenous *E6/E7* repression (Fig. 2E and Fig. 3). In specific, with the exception of miR-378f, the levels of nine of the ten selected miRNAs were modulated in the same direction (up or down) in both cell lines upon silencing of *E6/E7* expression, with five of these changes also exhibiting statistical significance in SiHa cells (Figs. 3B). Thus, although the two cell lines contain different HPV types and were established from cervical cancers with different histopathological backgrounds, there is a substantial similarity of the regulation of these miRNAs upon endogenous *E6/E7* inhibition.

### Effect of the p53 status on the regulation of *E6/E7*-modulated cellular miRNAs

The p53 protein can positively or negatively affect the expression of tumor-associated miRNAs [64]. Since p53 is strongly upregulated following *E6/E7* repression (Fig. 1B), we addressed the question whether the miRNA changes observed upon *E6/E7* silencing are p53-dependent. For these analyses, we comparatively investigated the miRNA regulation in parental HeLa cells and in “p53-null” HeLa cells. In the latter cells, endogenous p53 expression is silenced by a stably integrated vector expressing a short hairpin (sh)RNA that targets the p53 mRNA [65].

**Fig 4:**
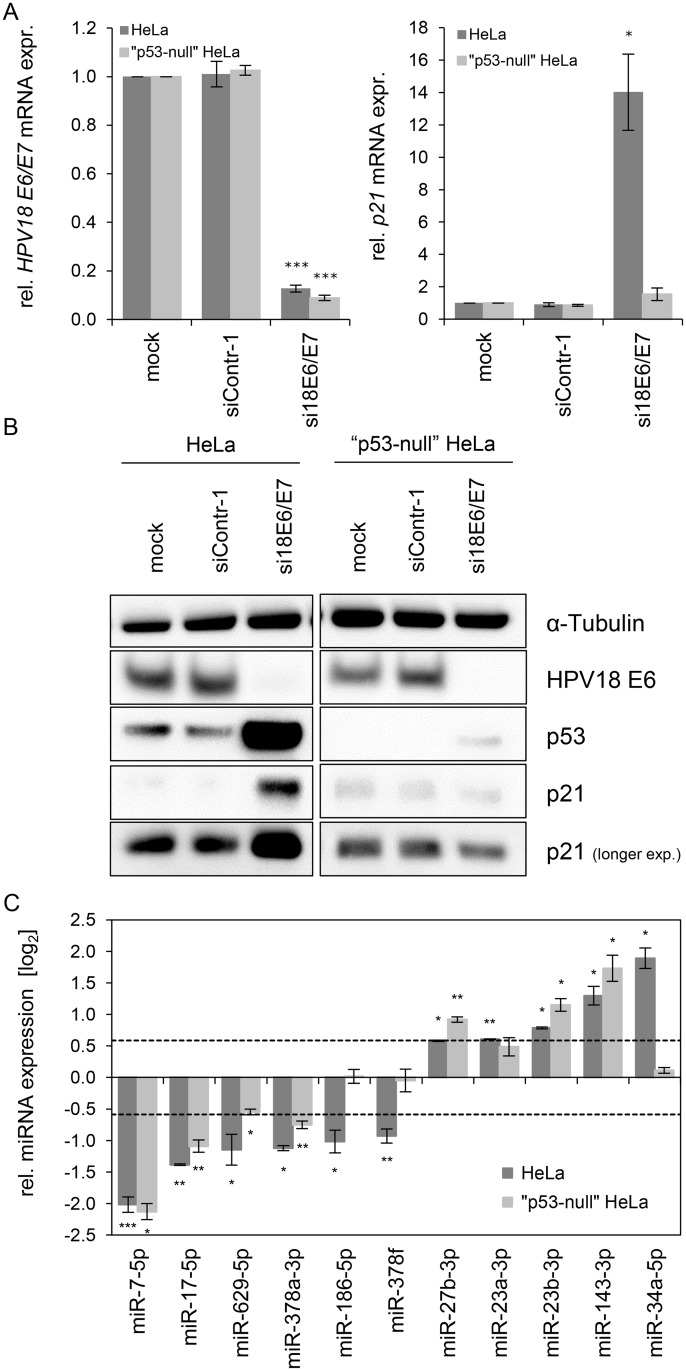
Effects of the p53 status on the *E6/E7*-dependent modulation of intracellular miRNAs. **(A)** qRT-PCR analysis of HPV18 *E6/E7* (left panel) and *p21* (right panel) mRNA expression, 72 h after transfection of parental or “p53-null” HeLa cells with si18E6/E7, control siRNA (siContr-1), or upon mock treatment. mRNA levels were normalized to *ACTB* and calculated relative to the mock control (mock). Data represent mean ± SEM (n = 3). Asterisks above columns indicate statistically significant differences from siContr-1-treated cells (p ≤ 0.05 (*), p ≤ 0.001 (***)). **(B)** Immunoblot analysis of HPV18 E6, p53 and p21 protein levels, 72 h after transfection of parental or “p53-null” HeLa cells with si18E6/E7 or siContr-1, or upon mock treatment. α-Tubulin: loading control. **(C)** qRT-PCR analyses of selected cellular miRNAs, 72 h after transfection of parental or “p53-null” HeLa cells with si18E6/E7 or siContr-1. miR-34a-3p, positive control miRNA (p53-inducible). Cellular miRNA levels were normalized to snRNA *RNU6–2* and calculated relative to siContr-1 (log_2_ display). Dashed lines: 1.5-fold up- or downregulation (log_2_(1.5) = 0.585). Data represent mean ± SEM (n = 3). Asterisks indicate statistically significant differences (p ≤ 0.05 (*), p ≤ 0.01 (**) and p ≤ 0.001 (***)).

HPV18 *E6/E7* expression could be repressed by siRNA in both cell lines with a comparable high efficiency (Fig. 4A, left panel). Basal p53 protein levels were undetectable in “p53-null” cells and remained extremely low even upon endogenous *E6/E7* silencing, indicating that the system exhibits only a very minute degree of leakiness (Fig. 4B). Consistently, expression of the p53 target gene *p21* is not increased upon *E6/E7* silencing in “p53-null” HeLa cells, both at the RNA and protein levels (Fig. 4A, right panel, and Fig. 4B), corroborating that the p53 pathway is efficiently blocked in these cells.

For miRNA analyses, we utilized miR-34a-5p as a positive control since it is well-documented as a p53-inducible miRNA [64]. In line, we found that miR-34a-5p amounts were also increased (6.2-fold) in the deep sequencing analysis upon *E6/E7* silencing, however, it did not reach the RPM threshold of > 1,000 (RPM_siContr-1_ = 64; RPM_si18E6/E7_ = 385). As shown in Fig. 4C, induction of miR-34a-5p upon *E6/E7* silencing is clearly detectable by qRT-PCR analyses in parental HeLa cells but virtually abolished in “p53-null” HeLa cells. In contrast to miR-34a-5p, the intracellular levels of eight of the ten miRNAs that were defined as *E6/E7*-regulated in parental HeLa cells also exhibited statistically significant changes in “p53-null” HeLa cells (Fig. 4C), with congruency in the direction of change (up or down). Only two miRNAs, miR-186–5p and miR-378f, showed a discrepant regulation in that they were no longer repressed in “p53-null” HeLa cells upon *E6/E7* silencing, suggesting that p53 may directly or indirectly reduce their expression. Taken together, these experiments indicate that a substantial proportion (eight of ten) of the most abundant and significantly affected cellular miRNAs is modulated by endogenous *E6/E7* expression in a p53-independent manner.

### HPV *E6/E7* increase intracellular levels of members of the oncogenic miR-17~92 cluster that reduce p21 expression in HPV-positive cancer cells

The 52 most abundant cellular miRNAs that were downregulated > 1.5-fold upon *E6/E7* silencing in both deep sequencing and qRT-PCR analyses encompassed miR-17–5p and miR-19b-3p, two members of the miR-17~92 cluster, and miR-93–5p, a member of the paralog miR-106b~25 cluster (Fig. 2D/E). Further qRT-PCR analyses revealed that all detectable additional members the miR-17~92 and miR-106b~25 clusters were also downregulated upon silencing of endogenous *E6/E7* expression (S3 Table). These findings indicate that continuous *E6/E7* expression increases the intracellular concentrations of multiple miRNAs from both oncogenic miRNA clusters in HPV-positive cancer cells.

Four miRNAs encoded by the miR-17~92 and miR-106b~25 clusters (miR-17–5p, miR-20a-5p, miR-106b-5p, miR-93–5p) are grouped into the miR-17 family, according to their identical seed sequence, and target two binding sites in the 3’ UTR of *p21* [66,67]. All four miRNAs exhibited a > 1.5-fold downregulation in HeLa cells upon endogenous *E6/E7* silencing in deep sequencing and/or qRT-PCR analyses (S3 Table). This raises the question whether the *E6/E7*-dependent increase of *p21*-targeting miRNAs may lead to *p21* repression in HPV-positive cancer cells and thereby contributes to their proliferative capacity and resistance towards senescence.

**Fig 5:**
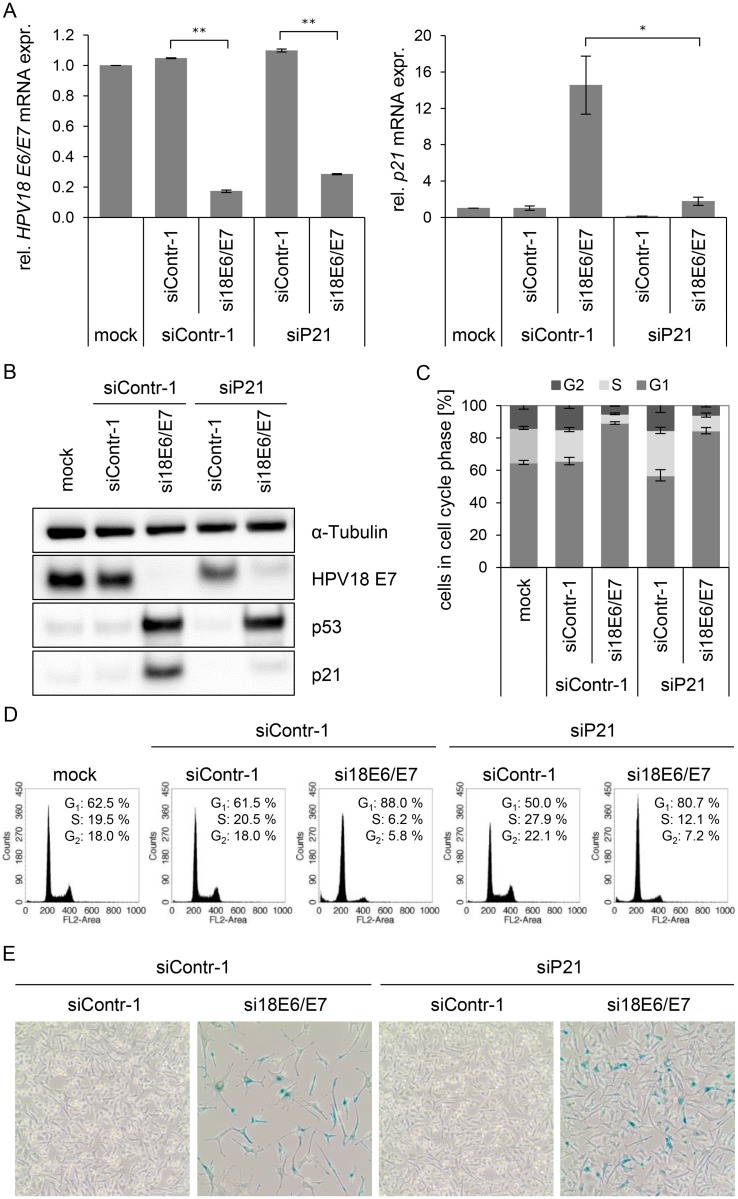
Influence of combined silencing of *p21* and HPV18 *E6/E7* expression on the senescent phenotype of HPV-positive cancer cells. **(A)** qRT-PCR analysis of HPV18 *E6/E7* (left panel) and *p21* (right panel) mRNA expression, 72 h after transfection of HeLa cells with the indicated siRNAs or in mock-treated cells. mRNA levels were normalized to *ACTB* and calculated relative to the mock control. Data represent mean ± SEM (n = 2 or 3). Asterisks above columns indicate statistically significant differences between the indicated treatments (p ≤ 0.05 (*), p ≤ 0.01 (**)). **(B)** Immunoblot analysis of HPV18 E7, p53, and p21 protein levels, 72 h after transfection of HeLa cells with the indicated siRNAs or upon mock-treatment. α-Tubulin: loading control. **(C + D)** Cell cycle distribution analyzed by FACS, 72 h after transfection of HeLa cells with the indicated siRNAs or upon mock treatment. Percentage of cells in the G_1_, S and G_2_ cell cycle phases are indicated. Representative samples of one experiment are shown as well as a summary of multiple biological replicates. Data represent mean ± SEM (n = 3). **(E)** HeLa cells were stained for expression of the senescence marker SA-β-Gal, 168 h after transfection with the indicated siRNAs. Visualization by bright field microscopy.

In order to investigate this possibility, we first addressed the controversially discussed issue whether p21 is required for senescence induction upon *E6/E7* silencing in HPV-positive cancer cells [13,68]. Therefore, siRNAs (si18E6/E7, siContr-1 and siP21) were utilized to achieve silencing of *E6/E7* only, *p21* only, or *E6/E7* together with *p21*. Inhibition of *p21* alone had no effect on HPV18 *E6/E7* expression (Fig. 5A, left panel), whilst it efficiently counteracted the induction of *p21* mRNA and protein levels upon *E6/E7* inhibition (Fig. 5A, right panel, and Fig. 5B). Cell cycle analyses revealed that *E6/E7* silencing alone led to an increase in G_1_- and decrease in S-phase populations, indicative for a G_1_-arrest (Fig. 5C/D). *p21* silencing alone, led to an increase in S-phase and reduction in G_1_-phase populations (Fig. 5C/D). Notably, the G_1_-arrest observed when inhibiting *E6/E7* alone was diminished when silencing *p21* in parallel, and S-phase populations doubled (from 6 to 12%, p-value = 0.059), 72 h post transfection. Moreover, 168 h after transfection only a fraction (15%) of the cells stained positive for the senescence marker Senescence-Associated β-Galactosidase (SA-β-Gal) when *p21* and *E6/E7* were silenced together, whereas the majority of cells remained unstained (Fig. 5E). This was in clear contrast to the results for *E6/E7* silencing alone, where almost all cells (85%) exhibited morphological signs of senescence (cellular enlargement and flattening, long cytoplasmic projections and positive staining for SA-β-Gal), 168 h after transfection (Fig. 5E). In conclusion, parallel silencing of *E6/E7* and *p21* strongly alleviated the induction of senescence that occurs upon mere *E6/E7* repression. This indicates that p21 is a contributor to the senescence induction upon *E6/E7* silencing in HPV-positive cancer cells.

**Fig 6:**
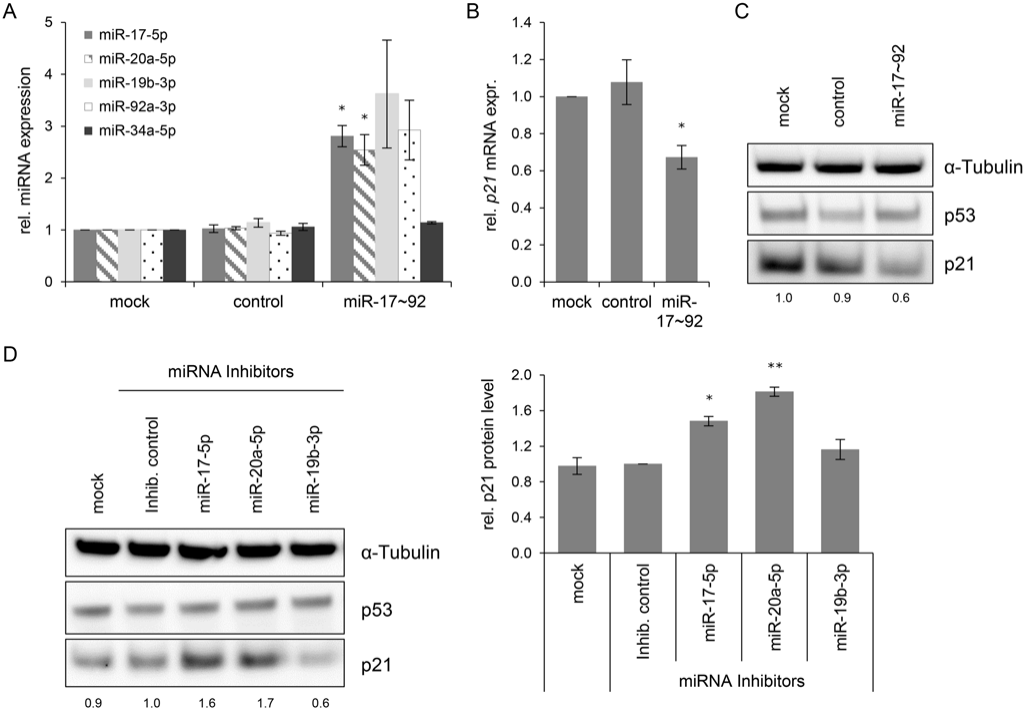
Effects of miRNAs of the miR-17~92 cluster on *p21* expression in HeLa cells. **(A)** qRT-PCR analyses of cellular miRNA levels, 72 h after transfection of HeLa cells with the indicated vectors or upon mock treatment. miR-17~92: vector coding for the *mir*-17~92 cluster; “control”: repective empty expression vector. miRNA levels were normalized to snRNA *RNU6–2* and calculated relative to the mock control. miR-17–5p, miR-20a-5p, miR-19b-3p, miR-92a-3p: encoded by the *mir*-17~92 expression vector; miR-34a-5p: negative control (not encoded by the vector). Data represent mean ± SEM (n = 3). Asterisks above columns indicate statistically significant differences from vector control-treated cells (p ≤ 0.05 (*)). **(B)** qRT-PCR analysis of *p21* mRNA expression, 72 h after transfection of HeLa cells with the indicated vectors or upon mock treatment. mRNA levels were normalized to *ACTB* and calculated relative to the mock control. Data represent mean ± SEM (n = 4). Asterisks above columns indicate statistically significant differences from vector control-treated cells (p ≤ 0.05 (*)). **(C)** Immunoblot analysis of p53 and p21 protein levels, 72 h after transfection with the indicated vectors. α-Tubulin: loading control. A representative image is shown with corresponding densitometrically quantified band intensities of p21, normalized to α-Tubulin and calculated relative to mock. **(D)** miRNA inhibitors against miR-17–5p and miR-20a-5p increase the expression of p21 in HeLa cells. Left panel: Immunoblot analysis of p53 and p21 protein levels, 72 h after transfection of HeLa cells with the indicated miRNA inhibitors, an inhibitor control (‘Inhib. control’), or upon mock treatment. α-Tubulin: loading control. A representative image is shown. Numbers below individual lanes correspond to densitometrically quantified band intensities for p21, normalized to α-Tubulin and calculated relative to the Inhib. control. Right panel: Summary of densitometric quantification of p21 protein signal intensities. Data represent mean ± SEM (n = 3). Asterisks above columns indicate statistically significant differences from Inhib. control-treated cells (p ≤ 0.05 (*), p ≤ 0.01 (**)).

Next, it was studied whether an experimental increase of *mir-*17~92 expression, encoding two *p21*-targeting miRNAs, miR-17–5p and miR-20a-5p [18], can contribute to keep basal levels of *p21* expression low in HeLa cells. Transfection of a *mir*-17~92 expression vector led to an increase of miR-17–5p, miR-20a-5p, miR-19b-3p and miR-92a-3p levels, as expected, but not of miR-34a-5p, which served as a negative control (Fig. 6A). Notably, basal *p21* expression in HeLa cells was reduced by overexpression of the miR-17~92 cluster, both at the mRNA and protein level (Fig. 6B/C).

In reciprocal experiments, we investigated whether the downmodulation of miR-17–5p and miR-20a-5p in HeLa cells might result in an upregulation of p21 levels. Therefore, HeLa cells were transfected with a control miRNA inhibitor (“Inhib. control”) that carries no homology to any known mammalian gene or with specific miRNA inhibitors of miR-17–5p, miR-20a-5p, and miR-19b-3p. The latter miRNA inhibitor served as additional control, since its target miRNA does not possess a known binding site in the *p21* transcript. The inhibitors of miR-17–5p and miR-20a-5p but neither the inhibitor control nor the miR-19b-3p inhibitor led to a significant upregulation of p21 protein levels (Fig. 6D). Taken together, these results indicate that continuous *E6/E7* oncogene expression in HPV-positive cancer cells is required to maintain miRNAs of the oncogenic miR-17~92 cluster at a level that keeps expression of the anti-proliferative p21 gene low.

### The *E6/E7*-dependent miRNA content of exosomes released from cervical cancer cells

**Fig 7:**
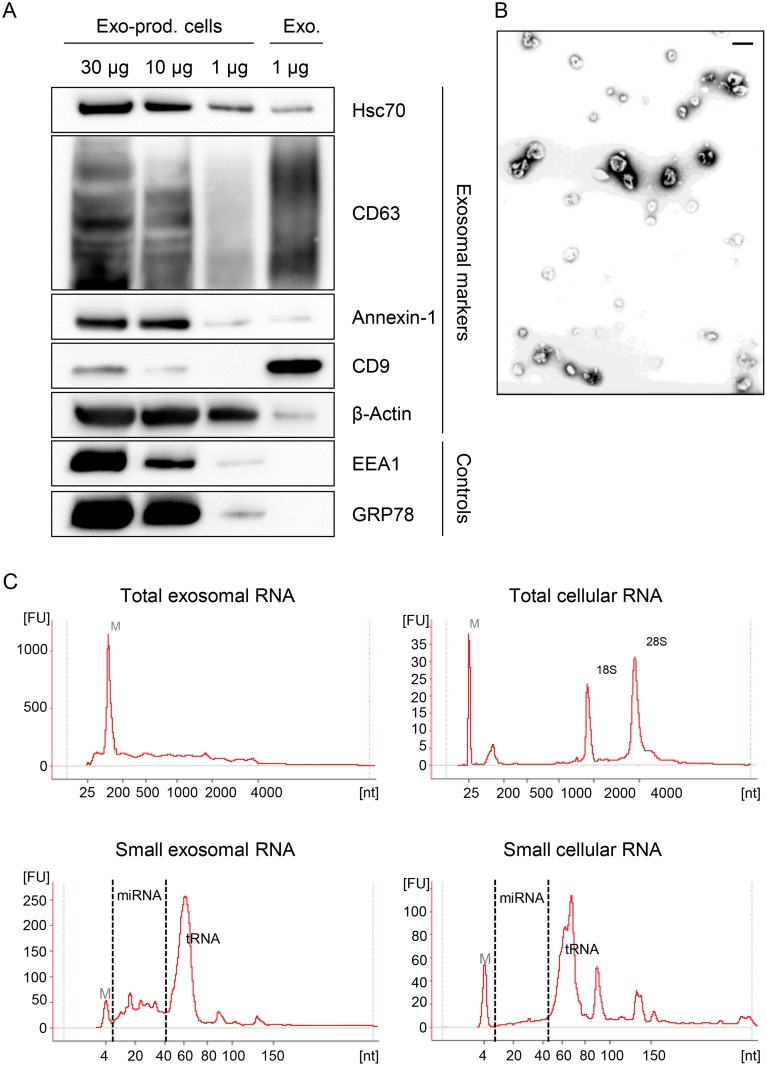
Characterization of exosomes secreted by HeLa cells used for small RNA deep sequencing. **(A)** Immunoblot analysis of total cellular extract (30, 10 and 1 μg) from exosome-producing cells, and of 1 μg protein from exosome preparations. Hsc70, CD63, Annexin-1, CD9 and β-Actin: exosomal markers; EEA1: early endosome marker; GRP78: ER marker. **(B)** Visualization of exosomes by electron microscopy. Bar corresponds to 100 nm. (C) Characterization of cellular and exosomal RNA. Electropherograms of total RNA isolated from HeLa cells and from RNAse A-treated exosomes. Upper panel: total RNA contents; lower panel: small RNA contents. M = marker. Shown are representative images for siContr-1-treated samples.

To investigate the influence of viral *E6/E7* expression on the exosomal miRNA contents, exosomes secreted by HeLa cells were isolated from the cell culture medium by employing a protocol for exosome enrichment involving sequential (ultra-)centrifugation steps [69], with minor modifications (see Material and Methods). A characterization of the exosome preparations used for the deep sequencing studies is presented in Fig. 7. The preparations stained positive for the exosomal markers HSC70, CD63, Annexin-1, β-Actin and CD9, showing the typical exosomal enrichment for the tetraspanins CD63 and CD9 [69] (Fig. 7A). The absence of detectable bands for the endoplasmatic reticulum (ER) marker GRP78 and the early endosome marker EEA1 indicate only minor, or no, contamination with vesicles from other origins. Electron microscopy (EM) revealed the presence of small membrane vesicles with a diameter of 50–100 nm, possessing the typical cup-shaped appearance of exosomes in EM analyses [69] (Fig. 7B).

To investigate possible effects of the HPV oncogenes on the miRNA composition of exosomes, we treated HeLa cells with siRNAs blocking endogenous HPV18 *E6/E7* expression or with control siRNA (siContr-1). Forty-eight hours after transfection, cells were allowed to secrete newly formed exosomes for 24 h into the cell culture medium pre-depleted of FBS-derived microvesicles. Inhibition of *E6/E7* expression upon transfection of synthetic siRNAs was maintained over the time period required for exosome production and secretion (Fig. 1). Total RNA was extracted from RNase A-treated exosomes and quality and quantity of the isolated RNA samples were assessed using an Agilent Bioanalyzer (Fig. 7C). In parallel, cellular RNA extracted from the respective exosome-producing cells was examined. The total RNA profile (Fig. 7C, upper panel) showed distinct differences between cellular and exosomal RNA, with exosomes lacking discernible 18S and 28S rRNA peaks, in agreement with previous publications [48,70]. Both cellular and exosomal RNA revealed a peak for transfer RNAs (tRNAs) and miRNAs (size range as indicated) in the small RNA profiles (Fig. 7C, lower panel).

**Fig 8:**
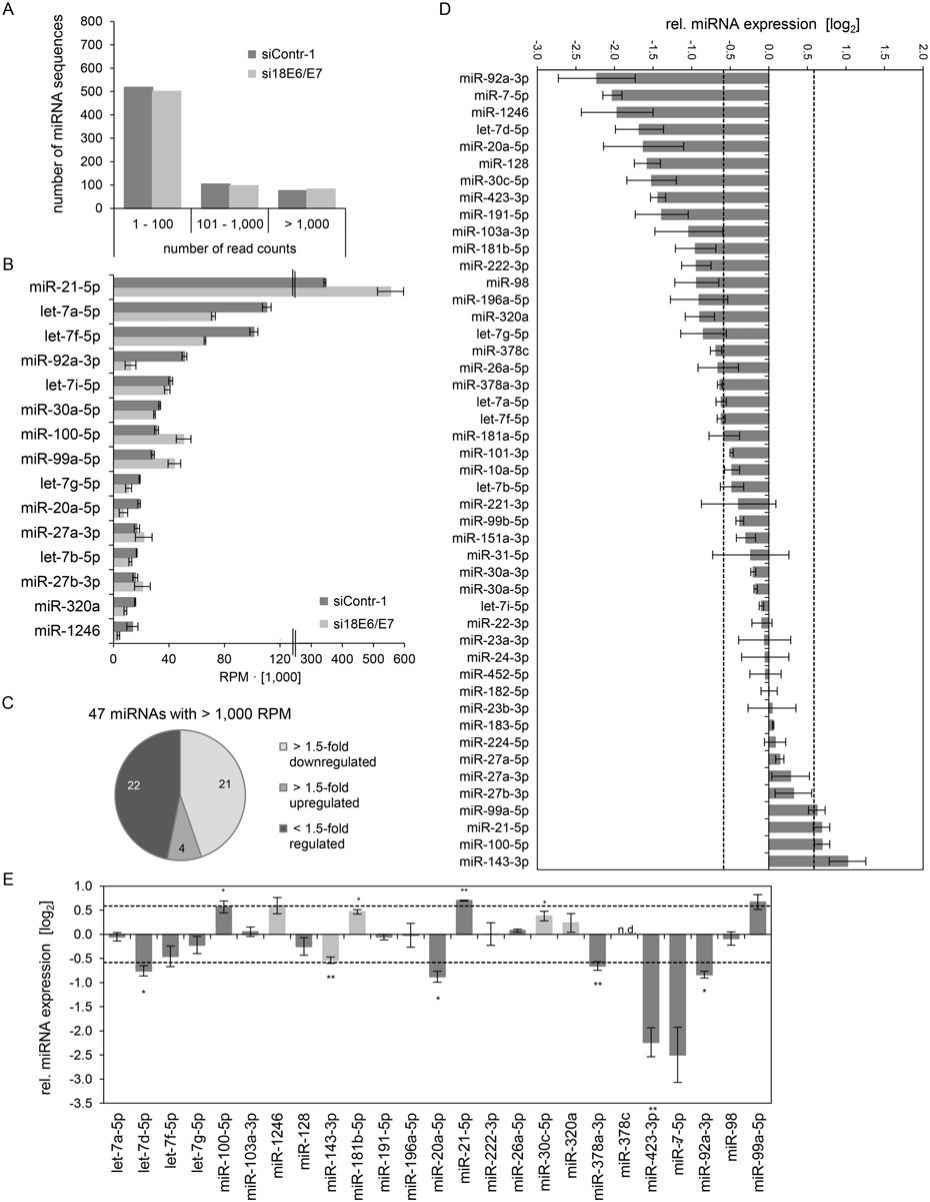
Inhibition of endogenous HPV18 E6/E7 expression: Effects on the miRNA composition of exosomes secreted by cervical cancer cells. Small RNA deep sequencing (A—D) and qRT-PCR analyses (E) of exosomal miRNAs, 72 h after transfection of HeLa cells with si18E6/E7 or control siRNA (siContr-1), and subsequent exosome purification from the cell culture supernatant. **(A)** Mean read count distribution of mature miRNA sequences in exosomes released from si18E6/E7- and siContr-1-treated HeLa cells (n = 3). Only miRNAs with a mean read count > 1 were considered. **(B)** The 15 most frequently sequenced exosomal miRNAs. Selection based on siContr-1 samples, respective values for si18E6/E7-treatment are indicated. Data represent mean ± SEM (n = 3). Interrupted x-Axis. **(C)** Overview on differentially deregulated (> 1.5-fold) exosomal miRNAs determined by small RNA deep sequencing. RPM values of si18E6/E7-treated samples were calculated relative to the control treatment (siContr-1). Only miRNAs with > 1,000 RPM in each sample were considered (n = 2). **(D)** Relative quantification of miRNAs in exosomes released from si18E6/E7- versus siContr-1-treated cells, as assessed by small RNA deep sequencing (log_2_ display). Dashed lines: 1.5-fold up- or downregulation (log_2_(1.5) = 0.585). Only miRNAs with > 1,000 RPM in each sample were considered. Data represent mean ± SEM (n = 3). **(E)** qRT-PCR analysis of *E6/E7*-dependent exosomal miRNAs identified by small RNA deep sequencing. Exosomal miRNA levels were normalized to miR-452–5p and miR-183–5p and calculated relative to siContr-1 (log_2_ display). Dashed lines: 1.5-fold up- or downregulation (log_2_(1.5) = 0.585). The column color shows regulation in the same (dark grey) or opposite (light grey) direction compared to the small RNA deep sequencing data of the individual miRNAs. Ct-values > 35 were considered as not detected (n.d.). Data represent mean ± SEM (n = 2 or 3). Asterisks indicate statistically significant differences (p ≤ 0.05 (*), p ≤ 0.01 (**)).

Subsequently, the exosomal RNA samples were converted into cDNA libraries, subjected to small RNA deep sequencing and initial analysis was performed as described above for cellular miRNAs. The composition of exosomal and intracellular small RNA fractions differed in that the relative percentage of miRNAs among different classes of small RNAs was approximately 50% lower in exosomes (S1 Fig). E6/E7 silencing only slightly affected the intracellular distribution of small RNA classes, but approximately doubled the relative percentage of miRNAs inside exosomes (S1 Fig). Mean read counts of exosomal sequences mapping to known human miRNAs ranged from 1 to 445,143 in exosomes (S1 Dataset, Fig. 8A for a read count distribution of exosomal miRNAs). RPM values of the 15 most frequently sequenced miRNAs in exosomes are displayed in Fig. 8B. Forty-seven exosomal miRNAs showed > 1,000 RPM in each sample and were subjected to further analysis. Thirty-six of these 47 exosomal miRNAs were also found among the 52 most abundant intracellular miRNAs with > 1,000 RPM (S1 Fig). Relative quantification of si18E6/E7- versus siContr-1-treated samples revealed that—among the 47 most commonly detected exosomal miRNAs—21 were down- and four upregulated more than 1.5-fold upon intracellular *E6/E7* silencing (Fig. 8C). The relative expression of each of these 47 miRNAs upon *E6/E7* silencing is displayed in Fig. 8D. The RPM_mean_ values of the 25 *E6/E7*-modulated exosomal miRNAs are indicated in S4 Table. They encompass several family members with identical seed regions, including the let-7 family (let-7a-5p, let-7d-5p, let-7f-5p, let-7g-5p), miR-378 family (miR-378a-3p, miR-378c), miR-99 family (miR-99a-5p, miR-100–5p), as well as members of the miR-17~92 cluster (miR-20a-5p, miR-92a-3p).

Next, the effects of *E6/E7* expression on exosomal miRNAs—as identified by small RNA deep sequencing—were validated by qRT-PCR analyses. So far, there is no common RNA species for normalization of exosomal miRNA levels available. Therefore, two miRNAs, miR-452–5p and miR-183–5p, were chosen as stable endogenous exosomal miRNA controls based on the small RNA deep sequencing data (in analogy to refs. [71,72]). Both miRNAs were frequently sequenced (> 1,000 RPM in each sample) and showed virtually no alterations of their exosomal concentrations upon *E6/E7* silencing versus control treatment (miR-452–5p: FC_mean_ = 0.99, miR-183–5p: FC_mean_ = 1.04).

Modulation (up or down) upon inhibition of HPV18 *E6/E7* expression was confirmed for roughly three quarters (72%) of the identified exosomal miRNAs by qRT-PCR analyses (Fig. 8E, dark grey columns). A statistically significant and > 1.5-fold decrease upon *E6/E7* silencing was detected for exosomal let-7d-5p, miR-20a-5p, miR-378a-3p, miR-423–3p, miR-7–5p, miR-92a-3p, whereas miR-21–5p exhibited a statistically significant and > 1.5-fold increase upon *E6/E7* silencing (illustrated in bold in S4 Table). These findings indicate that continuous HPV *E6/E7* oncogene expression determines a signature of seven miRNAs in exosomes secreted from HeLa cells in that it leads to significantly increased let-7d-5p, miR-20a-5p, miR-378a-3p, miR-423–3p, miR-7–5p, miR-92a-3p and decreased miR-21–5p levels.

**Fig 9:**
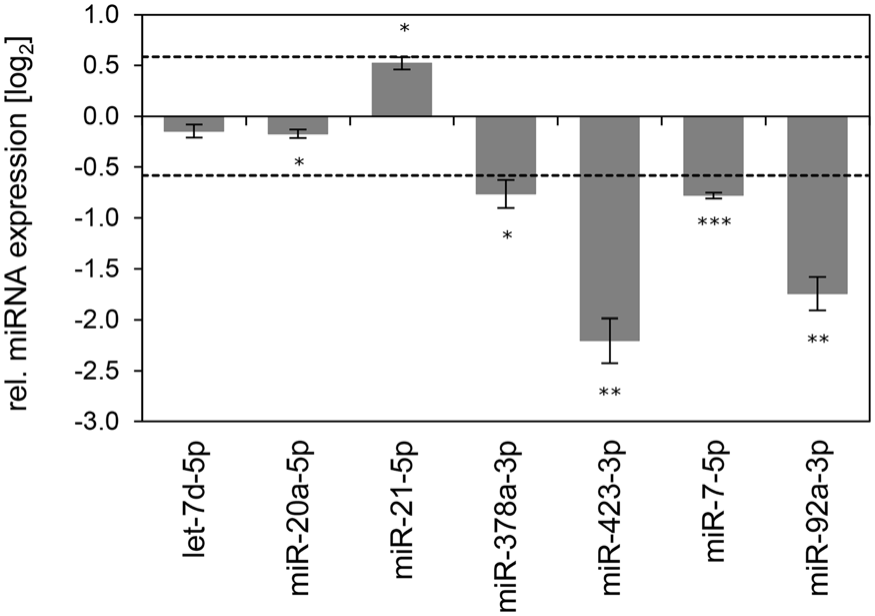
Inhibition of endogenous HPV16 *E6/E7* expression: Effects on selected exosomal miRNAs. qRT-PCR analysis of selected exosomal miRNAs, 72 h after transfection of SiHa cells with si16E6/E7 or control siRNA (siContr-1), and subsequent exosome purification from the cell culture supernatant. Exosomal miRNA levels were normalized to miR-452–5p and miR-183–5p and calculated relative to siContr-1 (log_2_ display). Dashed lines: 1.5-fold up- or downregulation (log_2_(1.5) = 0.585). Data represent mean ± SEM (n = 3). Asterisks indicate statistically significant differences (p ≤ 0.05 (*), p ≤ 0.01 (**) and p ≤ 0.001 (***)).

Comparative analyses of the above identified seven miRNAs in exosomes secreted by HPV16-positive SiHa cells revealed that the concentrations of all these are congruently modulated (up or down) upon inhibiting endogenous HPV16 *E6/E7* expression (Fig. 9). Six of these seven miRNA alterations were also statistically significant in SiHa cells, with a > 1.5-fold change observed for four of them (Fig. 9). Thus, similar to the results obtained for the regulation of intracellular miRNAs, there is substantial overlap in the *E6/E7-*dependent regulation of miRNA species in exosomes secreted by HPV16- and HPV18-positive cancer cells.

## Discussion

The growth of HPV-positive cancer cells requires the continuous expression of the viral *E6/E7* oncogenes [2,7,13,73–76]. To address the question whether this process is linked to alterations of the miRNA network, we here analyzed the influence of the *E6/E7* expression on the intracellular and exosomal miRNA pools of HPV-positive cancer cells. We found that ten of the 52 most abundant intracellular miRNAs identified by deep sequencing analyses of HeLa cells were significantly affected upon silencing of endogenous viral oncogene expression. Notably, they are enriched for miRNAs that are linked to the regulation of cell proliferation, senescence and apoptosis, suggesting that the *E6/E7*-linked modulation of the miRNA network contributes to the growth of HPV-positive cancer cells. Consistently, we observed that the sustained endogenous *E6/E7* expression is linked to an increase of miRNAs with growth promoting potential (e.g. members of the miR-17~92 cluster blocking p21 expression). In addition, we determine the miRNA content of exosomes secreted from HPV-positive cancer cells and delineate specific miRNAs whose exosomal concentrations are dependent on viral oncogene expression.

Several previous studies have identified miRNAs as potential targets for HPVs or have linked specific miRNAs to cervical carcinogenesis. By performing medline searches for the two keywords miRNA/microRNA and HPV/cervical cancer, 258 different publications were found (date: November 26^th^, 2014). Twenty-one of these reports encompass global miRNA profiling studies, performing analyses of the miRNA expression in tumorous versus normal cervical cancer tissue (13 publications), in different *in vitro* cell culture models (7 publications), or in both (1 publication). S5 Table provides an overview on these 21 studies and indicates the used platforms for miRNA analysis and the various experimental conditions. Only two of these studies performed comprehensive small RNA deep sequencing analyses [22,43], without a pre-selection of candidate miRNAs (e.g. for microarray design). We withdrew from these 21 publications the miRNAs that were reported to be differentially regulated in cervical cancer tissue or in *in vitro* models, and updated the original miRNA nomenclature of the publications to the current miRBase entries (release 21, June 2014). As a result, 483 different mature miRNAs have been proposed by these 21 studies to be HPV-dependent and/or deregulated in cervical cancer (S2 Dataset). Out of these 483 miRNAs, 201 were identified in more than one study, but showed substantial discordance with more than half of them (105 miRNAs) being regulated in opposite directions in different reports (S2 Dataset, S5 Table). Importantly, however, none of these experimental approaches have addressed the question whether the actual cellular miRNA composition of HPV-positive cancer cells depends on endogenous *E6/E7* expression. This is a critical issue since *E6/E7* expression levels are tightly controlled in HPV-positive cancer cells and it is not clear how this relates to the *E6/E7* levels obtained, for example, by ectopic *E6/E7* expression in keratinocytes. Furthermore, HPV-induced cell transformation requires additional alterations in the host cell, besides viral *E6/E7* expression. Thus, it is crucial to investigate the *E6/E7*-dependence of the cellular miRNA network by analyzing the effects of endogenous *E6/E7* expression levels, within the cellular background of HPV-transformed cervical cancer cells, in which the sustained *E6/E7* expression leads to the relevant cellular phenotype (maintenance of cell growth).

For these analyses, we chose HPV18-positive HeLa cells as a model for several considerations: *(i)* as common for HPV-positive cervical cancer cells, HeLa cells express the viral oncogenes from chromosomally integrated HPV copies, using the authentic *E6/E7* transcriptional promoter. *(ii)* HeLa cells mirror known critical mechanisms of HPV-linked carcinogenesis, such as inactivation of the p53 and pRb tumor suppressor proteins by the HPV E6 and E7 proteins, respectively. *(iii)* The growth of HeLa cells is strictly dependent on viral *E6/E7* expression [8,13,73,75,76] as is the case for primary cervical cancer cells freshly isolated from human tumor samples [74]. *(iv) E6/E7* silencing in HeLa cells results in the same phenotype as in primary cervical cancer cells, namely growth arrest and induction of cellular senescence [12–14,74,77]. *(v)* HeLa cells allow functional analyses within the intracellular milieu of an HPV-transformed cancer cell, which has acquired the necessary additional cellular alterations that are required for HPV-induced cervical carcinogenesis. Likely, these are not present in “normal” keratinocytes, which only in very rare instances are transformed to malignancy by the HPV *E6/E7* oncogenes alone [78]. *(vi)* A meta-analysis of mRNA transcriptome studies validated that RNAi-mediated silencing of endogenous *E6/E7* expression in HeLa cells [79] is one of the most suitable experimental approaches to predict molecular changes present in cervical cancer tissues [80].

We determined ten abundant intracellular miRNAs as *E6/E7*-dependent, based on our most stringent selection criteria (RPM values > 1,000; modulated > 1.5-fold upon *E6/E7* silencing in both deep sequencing and—statistically significant—in qRT-PCR analyses). Analyses in “p53-null” HeLa cells indicate that eight of the ten miRNAs are modulated upon *E6/E7* silencing in a p53-independent manner. These include miR-143–3p, the levels of which have been reported to be increased by p53 via enhanced post-transcriptional miRNA maturation [81]. However, our observation that miR-143–3p levels are very similarly regulated in parental and p53-deficient HeLa cells indicates that this mechanism is not responsible for the miR-143–3p increase upon endogenous *E6/E7* silencing.

We included the ten *E6/E7*-dependent miRNAs in S2 Dataset, resulting in a total number of 485 miRNAs identified in global miRNA expression analyses of cervical cancer biopsies and different *in vitro* cell culture models. Studies in cervical cancer biopsies reported alterations of six of the ten miRNAs that we identified here as being modulated by *E6/E7*. Notably, five of these six miRNAs exhibit congruent changes between our functional experiments *in vitro* and their expression patterns *in vivo* in at least one study, i.e. reduction upon *E6/E7* silencing in HeLa and upregulation in cervical cancer biopsies (miR-7–5p [43], miR-17–5p [27–30,34,37], miR-186–5p [35]) or increase upon *E6/E7* silencing in HeLa and downregulation in cervical cancer tissue (miR-23b-3p [28,37] and miR-143–3p [23,29,31,33,37,38,82]) (S2 Dataset). Only for miR-378a-3p, which was downregulated upon *E6/E7* silencing, our *in vitro* data contrasts the reported downregulation of this miRNA in cervical cancer tissue [28,34]. This high concordance with *in vivo* data provides further strong evidence for the suitability of the functional approach used here to identify *E6/E7*-dependent miRNA alterations.

Remarkably, multiple of the most abundant miRNAs found to be significantly affected by *E6/E7* silencing in HPV-positive cancer cells are known to be involved in the regulation of cell proliferation, senescence and apoptosis. Specifically, continuous *E6/E7* expression is necessary to maintain high intracellular levels of miR-7–5p, miR-629–5p, miR-378a-3p, miR378f, miR-17–5p, and miR-186–5p (S2 Table), which all have been linked to pro-tumorigenic activities. For example, miR-7–5p stimulated cell proliferation and tumorigenicity in lung cancer cells [83] and is linked to a more aggressive growth behavior of breast cancers [84]. miR-629–5p also promotes cell growth, has been found to be important for hepatocarcinogenesis via *HNF4a* repression [85], and targets the *NBS1* DNA tumor susceptibility gene [86]. Reduced miR-7–5p or miR-629–5p levels have also been both associated with cellular senescence [87]. Several members of the miR-378 family were among the most frequently sequenced miRNAs that decreased upon *E6/E7* silencing (S2 Table). miR-378a-3p can block the tumor-suppressive *Fus1* (*TUSC2*) and *SUFU* genes, leading to increased cell survival and tumor growth [88]. No functional data is yet available for miR-378f [89], however, it contains the same seed sequence as miR-378a-3p and therefore both miRNAs could regulate overlapping genes. miR-17–5p is discussed in more detail below. Finally, miR-186–5p is an inhibitor of the *FOXO1* tumor suppressor gene, which can exert anti-proliferative, pro-apoptotic and pro-senescent activities [90,91].

On the other hand, continuous *E6/E7* expression is linked to a decrease of the intracellular concentrations of miR-23a-3p, miR-23b-3p, miR-27b-3p, and miR-143–3p. The *E6/E7*-dependent reduction of miR-23a-3p is surprising since this miRNA often is elevated in cancers, suggesting that it acts pro-tumorigenic [92]. However, miR-23a-3p possesses pro-senescent potential [93,94] and has also been linked to apoptosis induction [92], indicating that miR-23a-3p can exert context-dependent pro- or anti-tumorigenic activities. miR-23b and miR-27b belong to the miR-23b-27b-24–1 cluster and both exhibit anti-tumorigenic activities [95,96]. Finally, among the 52 most commonly sequenced miRNAs, miR-143–3p represents the most strongly activated miRNA after *E6/E7* silencing. It acts anti-proliferative [97], including in cervical cancer cell lines [34], and an increase of miR-143–3p levels has been linked to senescence [97]. It is also remarkable that three of the four abundant miRNAs that are significantly decreased by sustained *E6/E7* expression are associated with the metabolic alterations typical for cancer cells. Specifically, lowered levels of miR-23a-3p and miR-23b-3p have been linked to enhanced glutamine catabolism [98] and a decrease of miR-143–3p favors glucose metabolism by aerobic glycolysis (Warburg effect) [99].

**Fig 10:**
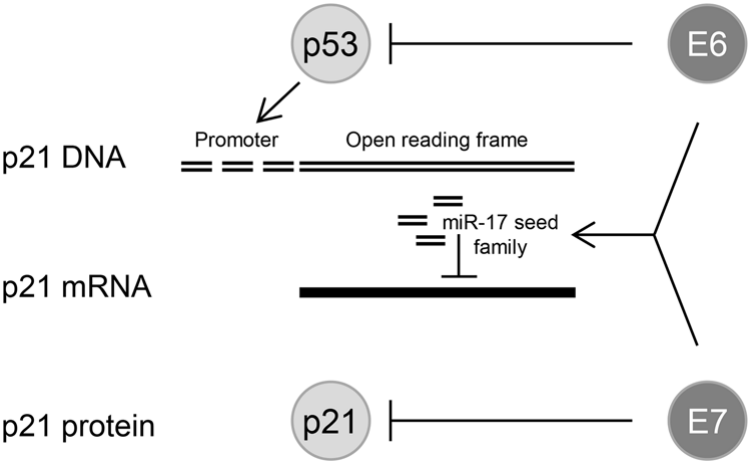
HPV oncogenes control *p21* expression at multiple levels. E6 can repress *p21* transcription at the promoter level by inducing the degradation of the *p21* transcriptional activator p53; sustained *E6/E7* expression maintains the concentration of miR-17 family members in HPV-positive cancer cells which repress *p21* expression by targeting the *p21* mRNA; the E7 protein can directly bind to the p21 protein and inhibit its function.

The identification of cellular miRNAs in the present work that are dependent on sustained endogenous *E6/E7* expression forms a basis for future functional studies. Here, we took a closer look at members of the tumorigenic miR-17~92 cluster, since *(i)* miR-17–5p was among the top ten hits of abundant miRNAs of which the expression was maintained by the *E6/E7* oncogenes, *(ii)* all other members of this cluster, as well as of the paralog cluster miR-106b~25, were also downregulated by *E6/E7* silencing when applying less stringent selection criteria (S3 Table), *(iii)* several members of the miR-17~92 cluster are well-decumented to be overexpressed in cervical cancer tissues, including the tested miR-17–5p [27–30,34,37] and miR-20a-5p [23,30,31,34,35] (also see S2 Dataset), and *(iv)* four of these miRNAs (miR-17–5p, miR-20a-5p, miR-93–5p, and miR-106b-5p) possess the same seed sequence and can bind to the 3’ UTR of the *p21* mRNA [18]. Our finding that the concomitant inhibition of *p21* and *E6/E7* expression led to an alleviation of the senescent phenotype, compared to cells in which only *E6/E7* was repressed, supports the notion that *p21* contributes to the induction of cellular senescence upon *E6/E7* inhibition in HPV-positive cancer cells [13]. In view of the strong anti-proliferative and pro-senescent potential of *p21*, it seems critical for the growth of HPV-positive tumor cells to block p21 function. Our findings that miR-17–5p and miR-20a-5p inhibitors significantly induced endogenous p21 protein levels, indicates that oncogenic HPVs reduce *p21* expression in cervical cancer cells by increasing the intracellular concentrations of members of this miRNA seed family. This provides evidence for a third layer of negative regulation of *p21* by the HPV oncogenes, in addition to interfering with p53-mediated transcriptional *p21* activation via E6-mediated p53 degradation [100] and the inhibitory E7/p21 protein/protein interaction [101,102] (Fig. 10).

Comparative analyses of the ten HPV18 *E6/E7*-dependent cellular miRNAs (HeLa) in HPV16-positive cells (SiHa) revealed a substantial overlap in their regulation patterns upon inhibition of endogenous *E6/E7* expression. This is not necessarily expectable since the two cell lines are derived from a cervical adenocarcinoma and a squamous cell carcinoma, respectively, and the miRNA composition of tumor cells can substantially vary even for the same cancer form, dependent on the histological background or differentiation status [103,104]. Since HPV16 and HPV18 *E6/E7* share most of their functions, the overlap in miRNA regulation across tumor cells of different histopathological origin provides further evidence for its *E6/E7*-dependence.

The present work also represents the first study investigating global changes of the miRNA composition of exosomes released from HPV-positive cancer cells, in dependence on endogenous viral oncogene expression. Consistent with the view that exosomal sorting of miRNAs is a directed process [105], the most commonly sequenced intracellular and exosomal miRNAs exhibited only a partial overlap. We also observed that E6/E7 silencing increases the percentage of exosomal miRNAs relative to other small RNA fractions, raising the possibility that the viral oncogenes affect exosomal sorting of small RNAs. Among the 47 most frequently sequenced exosomal miRNAs, 25 were modulated > 1.5-fold by silencing *E6/E7* expression. Seven of those also exhibited statistical significance in the validating qRT-PCR analyses.

Our results show that continuous *E6/E7* expression is linked to an upregulation of let-7d-5p, miR-20a-5p, miR-378a-3p, miR-423–3p, miR-7–5p, miR-92a-3p and a downregulation of miR-21–5p, in exosomes secreted from HeLa cells. Interestingly, several of these miRNAs exert cancer-associated activities inside cells. Let-7d-5p belongs to the let-7 miRNA family, which is considered to primarily act tumor-suppressive [106]. However, specific analyses of the let-7d family member also indicate anti-apoptotic activity by targeting the 3’ UTR of *caspase 3* [107] and a strong increase of let-7d-5p levels has been observed during progression of breast cancers [108]. The other five of the six abundant exosomal miRNAs that are maintained by continuous *E6/E7* expression have been primarily linked to pro-tumorigenic activities. The pro-oncogenic potential of miR-7–5p and miR-378a-3p is discussed above. miR-20a-5p and miR-92a-3p are both members of the miR-17~92 cluster. miR-20a-5p can block oncogene-induced senescence via *p21* repression [109], whereas miR-92a-3p possesses anti-apoptotic potential [110]. miR-423–3p has been shown to promote G_1_/S transition and cell growth. On the other hand, miR-21–5p, which is considered to be pro-tumorigenic [111], was the only miRNA among the 47 most frequently sequenced miRNA species in exosomes that was significantly upregulated > 1.5-fold upon *E6/E7* silencing, indicating that continuous *E6/E7* expression is associated with reduced exosomal miR-21–5p levels. Thus, taken together, with the exception of miR-21–5p, sustained *E6/E7* expression in HPV-positive cancer cells is linked to exosomal miRNA alterations that possess known pro-proliferative or anti-apoptotic potential. These findings complement results indicating that endogenous *E6/E7* expression in HPV-positive cancer cells is also linked to exosomal protein alterations with growth promoting and anti-apoptotic potential, e.g. upregulation of Survivin [77]. Comparative analyses of the seven miRNAs in exosomes secreted by HPV16-positive SiHa cells revealed a substantial overlap in their modulation upon endogenous *E6/E7* silencing. Thus, as observed for intracellular miRNAs, there is a similar regulation of *E6/E7*-dependent miRNAs in exosomes secreted by tumor cells that contain different HPV types and that are of different histological origin.

Our observations concerning exosomal miRNA contents could be relevant for intercellular communication in that HPV-positive cells might convey a tumor-promoting message to surrounding cells via exosomes, as has been reported for two of the *E6/E7*-dependent exosomal miRNAs, miR-92a-3p [55] and miR-378a-3p [52]. Of note, however, studies on the intercellular communication via exosomes have to critically consider technical limitations that are still unresolved. Despite an increasing number of examples showing that an intercellular crosstalk via exosomal miRNAs is possible in cell culture, the physiological significance of these observations is often uncertain [47,112]. Specifically, most studies utilized concentrated exosome preparations and it is not clear how these experimental conditions relate to exosome concentrations in the physiological context [113] which are very low in biological fluids (within 100 fM range). This might be below the threshold for exerting significant physiological effects *in vivo* [114,115] since it has been estimated that miRNAs require intracellular levels of greater than 1,000 copies per cell to trigger measurable activity on their mRNA targets [115]. These questions could be addressed once experimental systems to test the physiological relevance of exosomes become available, which is a topic of intense ongoing research in the exosome field [47,112].

The identification of an *E6/E7*-dependent miRNA signature in exosomes may also bear diagnostic potential. Circulating miRNAs are currently intensively investigated as new, minimally invasive biomarkers for early diagnosis, prognosis and prediction of response to specific therapies [116,117]. A significant source of miRNAs in extracellular fluids, like serum or saliva, are exosomes [118]. Major advantages of using exosomal miRNAs as biomarkers include their high stability and the possibility to increase the sensitivity of miRNA amplification from human biologic fluids by exosome isolation and enrichment [71,118]. It thus will be interesting to investigate whether the *E6/E7*-dependent miRNA changes identified here are mirrored in exosomes isolated from body fluids of patients suffering from HPV-linked diseases, such as in the serum, cervical lavages of cervical cancer patients or saliva of head and neck cancer patients.

Taken together, this study shows that the endogenous *E6/E7* expression in HPV-positive cancer cells is linked to increased concentrations of multiple pro-proliferative, anti-senescent and anti-apoptotic miRNAs, while the amounts of anti-proliferative, pro-senescent and pro-apoptotic miRNAs are reduced. This applies to abundant miRNA species both inside the cell and in exosomes. These findings imply that the viral *E6/E7* oncogenes affect the growth of HPV-positive cancer cells by manipulating the intracellular and exosomal miRNA compositions. It will be interesting for future studies to further decipher the role of these miRNAs for the proliferation and survival of HPV-positive cancer cells. Moreover, since therapeutic agents acting on the miRNA level are now entering the clinic [117,119] and since therapeutically useful E6/E7 inhibitors are still not available, it will be important to evaluate whether a correction of the *E6/E7*-dependent miRNA alterations by miRNA mimics or inhibitors possesses therapeutic potential for the treatment of HPV-linked premalignant and malignant lesions.

## Materials and Methods

### Cell culture, transfections and treatment conditions

HPV18-positive HeLa (obtained from the tumor bank of the German Cancer Research Center, Heidelberg) and HPV16-positive SiHa cervical carcinoma cells (obtained from the American Tissue Culture Collection, ATCC) were cultured in DMEM (Gibco, Life Technologies, Carlsbad, CA, USA) containing 5% fetal bovine serum (Gibco, Life Technologies), 2 mM L-glutamine, 100 U/ml penicillin, 100 μg/ml streptomycin (Sigma-Aldrich, Saint Louis, MO, USA). “p53-null” HeLa cells were described in detail in ref. [65].

Synthetic siRNAs (Ambion, Life Technologies, Carlsbad, CA, USA) were transfected with DharmaFECT I (Thermo Fisher Scientific, Waltham, MA, USA), according to the manufacturer’s instructions, to reach a final siRNA concentration of 10 nM. For silencing viral HPV18 or HPV16 *E6/E7* oncogene expression, three different siRNAs, which each target all three HPV *E6/E7* transcript classes, were generated [8]. To minimize potential off-target effects [120,121] the three siRNAs were pooled at equimolar concentrations (referred to in the text as “si18E6/E7” or “si16E6/E7”, respectively). The siRNA target sequences were as follows: HPV18 *E6/E7–*1 5’-CCACAACGUCACACAAUGU-3’; HPV18 *E6/E7–*2 5’-CAGAGAAACACAAGUAUAA-3’; HPV18 *E6/E7–*3 5’-UCCAGCAGCUGUUUCUGAA-3’, HPV16 *E6/E7–*1 5’-CCGGACAGAGCCCAUUACA-3’; HPV16 *E6/E7–*2 5’- CACCUACAUUGCAUGAAUA-3’; HPV16 *E6/E7–*3 5’- CAACUGAUCUCUACUGUUA-3’; *p21 (CDKN1A)* 5’-CAAGGAGUCAGACAUUUUA-3’. Control siRNA “siContr-1”, 5′-CAGUCGCGUUUGCGACUGG-3′, contains at least four mismatches to all known human genes.

miRNA Inhibitors (Qiagen, Hilden, Germany) and the miScript Inhibitor Negative Control (Qiagen) were transfected with DharmaFECT I (Thermo Fisher Scientific), according to the manufacturer’s instructions, to reach a final concentration of 100 nM for miRNA inhibitors.

Plasmids were transfected by calcium phosphate co-precipitation as described by Chen and Okayama [122]. The plasmid expressing the *mir*-17~92 cluster (pcDNA3.1/V5-His-TOPO-mir17~92, [123]) was a gift from Joshua Mendell (Addgene plasmid # 21109) and the pcDNA3.1 empty vector (Life Technologies) was used as negative control.

### Cell cycle analyses

For cell cycle analysis, cells were trypsinized 72 h after transfection, washed in ice-cold PBS and fixed in 80% cold ethanol overnight at -20°C. Subsequently cells were pelleted, resuspended in phosphate buffered saline (PBS, 137 mM NaCl, 2.7 mM KCl, 4.3 mM Na_2_HPO_4_, 1.4 mM KH_2_PO_4_, pH 7.4) containing 1 mg/ml RNase A (Roche Diagnostics) and 25 μg/ml propidium iodide (Sigma-Aldrich) and incubated for 30 min at room temperature (RT). Cell cycle analyses were performed by fluorescence-activated cell sorting (FACS) using a FACSCalibur Flow Cytometer (BD Biosciences, Heidelberg, Germany) with CellQuest Pro software provided by the manufacturer. Quantitation of the percentage of cells in the individual phases was performed using FlowJo software (Tree Star, Ashland, OR, USA), applying the Dean-Jett-Fox model [124].

### Senescence assay

HeLa cells were plated on glass cover slips, followed by transfection with siRNAs, as described above. Staining for senescence-associated β-galactosidase (SA-β-Gal) activity was performed 168 h after transfection, as described by Dimri *et al*. [125].

### Generation and purification of exosomes

For exosome production cells were plated on 15 cm dishes to reach 80% confluence 72 h post transfection. Forty-eight h post transfection the cells were washed with DMEM and cultured for 24 h in “vesicle-depleted medium” (complete medium depleted of FBS-derived microvesicles by overnight centrifugation at 100,000 g). The conditioned medium was collected and cleared from intact cells and cellular debris by three rounds of centrifugation at 300 g, 3,000 g, and 10,000 g for 20 min. Subsequently, exosomes were pelleted from the resulting supernatant by ultracentrifugation at 100,000 g for 70 min using a SW28 rotor (Beckman Coulter, Fullerton, CA, USA), resuspended in 36 ml PBS, and re-centrifuged at 100,000 g for 70 min. All centrifugation steps were performed at 4°C. The final pellet was resuspended in 100 μl PBS and an aliquot was analyzed by electron microscopy. For each preparation, the total protein concentration was quantified using the Qubit Protein Assay (Life Technologies). The corresponding exosome-producing cells were harvested and pelleted by centrifugation at 800 g for 3 min, resuspended and washed in 800 μl PBS, and re-pelleted.

### Immunoblotting

Cell pellets were lysed in RIPA buffer (10 mM Tris (pH 7.5), 150 mM NaCl, 1 mM EDTA, 1% Nonidet P_40_, 0.5% Na-Deoxycholat, 0.1% SDS, supplemented with 25 μl/ml Pefabloc (Merck, Whitehouse Station, NJ, USA) and 10 μl/ml of Protease Inhibitor Cocktail (Sigma-Aldrich)) for 30 min on ice and proteins were collected by centrifugation at 12,000 g for 15 min. Protein concentrations were determined using the Bio-Rad Protein Assay (Bio-Rad, Hercules, CA, USA), employing bovine serum albumin as standard. For Western blot analysis, protein extracts and exosome samples were boiled in SDS sample buffer (for reducing conditions: 8% SDS, 250 mM Tris-HCL (pH 6.8), 20% β-mercaptoethanol, 40% glycerol, 0.008% bromphenol blue; for non-reducing conditions without β-mercaptoethanol) for 5 min at 95°C and separated on NuPAGE Novex 4–12% Bis-Tris Mini Gels (Life Technologies). Proteins were electrotransferred onto an Immobilon-P membrane (Millipore, Bedford, MA, USA) using the Trans-Blot Semi-Dry Transfer Cell (Bio-Rad). Membranes were blocked with 5% skim milk powder (Saliter, Obergünzburg, Germany) in PBS-T (PBS supplemented with 0.1% Tween-20) for 1 h at RT. Membranes were probed with primary antibodies over night at 4°C in PBS-T, followed by incubation with the respective HRP-conjugated secondary antibody in PBS-T for 1 h at RT. Proteins were visualized using the ECL Prime Western Blotting Detection Reagent (GE Healthcare, Buckinghamshire, UK). Images were acquired using the Fusion SL Gel Detection System (Vilber Lourmat, Marne-la-Vallée, France), band densities were determined by BioID image analysis software (Vilber Lourmat).

The following primary antibodies were used: mouse anti-α-Tubulin (Merck), CP06, dilution 1:5,000; mouse anti-β-Actin (Sigma-Aldrich), A2228, 1:10,000; mouse anti-Annexin 1 (BD Transduction, Heidelberg, Germany), #610066, 1:10,000; mouse anti-CD63 (Santa Cruz Biotechnology, Santa Cruz, CA, USA), sc-5275, 1:400 at non-reducing conditions; mouse anti-CD9 (BD Pharmingen, San Diego, Ca, USA), #555370, 1:200 at non-reducing conditions; mouse anti-GRP78 (BD Transduction), #610979, 1:1,000; chicken anti-HPV18 E7 (E7C) [126]; mouse anti-HPV18 E6 (Arbor Vita Corporation Sunnyvale, CA, USA) AVC #399; mouse anti-HPV16 E7 (NM2, kind gift of Dr. Martin Müller, German Cancer Research Center, Heidelberg, Germany); mouse anti-HPV16 E6 (Arbor Vita Corporation Sunnyvale, CA, USA) AVC #843; rat anti-Hsc70 (Stressgen, San Diego, CA, USA), ADI-SPA-815, 1:1,000; mouse anti-p53 (BD Pharmingen), #554293, 1:500; mouse anti-EEA1 (BD Transduction Laboratories), #E41120, 1:2,000; mouse anti-pRb (Cell Signaling), #9309, 1:1,000; rabbit anti-phospho(Ser807/811)-pRb (Cell Signaling), #9308, 1:1,000; rabbit anti-CyclinA1 (Santa Cruz Biotechnology), 1:2,000. The following HRP-conjugated secondary antibodies were applied: anti-mouse IgG (Promega, Madison, WI, USA), W4021, 1:5,000; anti-chicken IgY (Promega), G1351, 1:2,500; anti-rat IgG (Dianova, Hamburg, Germany), #112035003, 1:5,000; and anti-goat IgG (Promega), V8051, 1:3,000.

### Electron Microscopy (EM)

Purified exosomes (6 μl, corresponding to 1 to 4 μg protein, depending on the experiment) were layered onto carbon-coated copper grids (300 mesh, Plano, Wetzlar, Germany) and allowed to dry at RT. Grids were then washed with water for 5 min and stained with 2% uranyl acetate in water (Polysciences, Warrington, PA, USA) for 30 sec to 1 min. Imaging was performed at an acceleration voltage of 80 kV with the EM10 Electron Microscope (Zeiss, Oberkochen, Germany).

### RNA extraction, quantification and quality determination

Total cellular RNA, including miRNA, was isolated with the miRNeasy Mini Kit (Qiagen) following the manufacturer’s protocol. All optional washing steps were included and RNA was eluted in a final volume of 30 μl RNase-free water. Total exosomal RNA, including miRNA, was isolated using the protocol described for cells with slight modifications: Exosome samples were pre-treated with 100 ng/μl RNAse A (Roche) for 30 min at 37°C, immediately before extracting RNA. Prior to the addition of chloroform and phase separation, 12 μg glycogen from *Mytilus edulis* (Sigma) were added to the sample. RNA concentrations were measured with the NanoDrop ND-1000 at 260 nm. RNA quality was assessed with the Agilent 2100 Bioanalyzer (Agilent Technologies, Böblingen, Germany) using the Agilent RNA 6000 Pico Kit (total RNA) and the Agilent Small RNA Kit (small RNA). The 2100 Bioanalyzer Expert Software B.02.08. (Agilent) was applied to generate electropherograms. RNA samples were stored at -80°C until further analysis.

### mRNA Quantitative Reverse Transcription-PCR (qRT-PCR)

For mRNA analysis, reverse transcription of 1 μg total RNA was carried out with the ProtoScript First Strand cDNA Synthesis Kit (NEB) according to the manufacturer’s instructions, using oligo-dT primers in an end volume of 20 μl. To assess for genomic DNA contamination of the sample, a no reverse transcriptase control (RT-) was prepared for each experiment by replacing the M-MuLV Enzyme Mix with RNase-free H_2_O. qRT-PCR reactions were performed with the SYBR Green PCR Master Mix (Applied Biosystems) and a final primer concentration of 500 nM on a 7300 Real-Time PCR System Detector (Applied Biosystems). Two μl of a 1:5 dilution of the original cDNA were used for each reaction and samples were run in triplicate for each experiment. A no template control (NTC) to monitor contamination of the reagents was included for each primer pair by adding H_2_O instead of cDNA template. The forward (fwd) and reverse (rev) primer sequences (Eurofins MWG, Ebersberg, Germany) used for determining mRNA expression levels were as follows: HPV18 *E6/E7* fwd 5’-ATGCATGGACCTAAGGCAAC-3’, HPV18 *E6/E7* rev 5’-AGGTCGTCTGCTGAGCTTTC-3’, HPV16 *E6/E7* fwd 5’-CAATGTTTCAGGACCCACAGG-3’, HPV16 *E6/E7* rev 5’-CTCACGTCGCAGTAACTGTTG-3’, *p21 (CDKN1A) fwd* 5’-GACCATGTGGACCTGTCACT-3’, *p21 (CDKN1A) rev* 5’-GCGGATTAGGGCTTCCTCTT-3’, *ACTB* fwd 5’-AGACAGTATACCCCATGCTGCAT-3’, *ACTB* rev 5’-TCCAATGTGTCTCCATACACAGA-3’. Cycling conditions have been previously described [127]. The sizes of the PCR products were initially analyzed by agarose gel electrophoresis and subsequently checked by melting point analysis after each reaction using the 7300 System SDS Software (Applied Biosystems). Cycle thresholds (Ct) were normalized to the Cts of *ACTB* using the comparative Ct (2^-ΔΔCt^) method [128]. Fold enrichments were calculated as compared to the values from the mock control.

### miRNA qRT-PCR

miRNA expression was detected using the miScript PCR System (Qiagen) with miRNA specific primers according to the manufacturer’s instructions. Briefly, 1 μg cellular RNA was converted to cDNA using the miScript II Reverse Transcription Kit (Qiagen) in a reaction volume of 20 μl using 5x miScript HiFlex Buffer. Due to the low yield of exosomal RNA and consequently the lack of accurate RNA quantification, identical volumes of exosomal RNA (12 μl) were used for cDNA synthesis. An RT- control was prepared for each experiment. qRT-PCR reactions were performed with the miScript SYBR Green PCR Kit (Qiagen) on a 7300 Real-Time PCR System Detector (Applied Biosystems) using Qiagen’s recommended cycling conditions. Two μl of a 1:100 dilution of the original cDNA were used for each reaction and samples were run in triplicate for each experiment. To monitor contamination of the reagents, an NTC was included for each primer pair. Data were analyzed using the comparative Ct (2^-ΔΔCt^) method [128] with the small nuclear RNA *RNU6–2* as endogenous control for cellular samples and respective mock- or control-treated samples (as indicated) as reference. Normalization of exosomal samples was conducted against an average of the expression of miR-452–5p and miR-183–5p. These two miRNAs were chosen as endogenous exosomal miRNAs for normalization since they were among the most frequently sequenced exosomal miRNAs in HeLa cells (> 1,000 RPM in each sample) and showed virtually no regulation in exosomes upon silencing of HPV18 *E6/E7* versus control treatment based on the deep sequencing data (miR-452–5p: FC_mean_ = 0.99, miR-183–5p: FC_mean_ = 1.04). miRNAs with Ct values > 35 were below detection limit and excluded from analysis. The following miScript Primer Assays (Qiagen) were applied: Hs_let-7a_2 (hsa-let-7a-5p), Hs_let-7d_1 (hsa-let-7d-5p), Hs_let-7f_1 (hsa-let-7f-5p), Hs_let-7g_2 (hsa-let-7g-5p), Hs_miR-100_2 (hsa-miR-100–5p), Hs_miR-103a_1 (hsa-miR-103a-3p), Hs_miR-106b_1 (hsa-miR-106b-5p), Hs_miR-1246_2 (hsa-miR-1246), Hs_miR-125a_1 (hsa-miR-125a-5p), Hs_miR-128_1 (hsa-miR-128), Hs_miR-1307_1 (hsa-miR-1307–3p), Hs_miR-143_1 (hsa-miR-143–3p), Hs_miR-17_2 (hsa-miR-17–5p), Hs_miR-181b_1 (hsa-miR-181b-5p), Hs_miR-182_2 (hsa-miR-182–5p), Hs_miR-183_2 (hsa-miR-183–5p), Hs_miR-186_1 (hsa-miR-186–5p), Hs_miR-191_1 (hsa-miR-191–5p), Hs_miR-196a_2 (hsa-miR-196a-5p), Hs_miR-19b_2 (hsa-miR-19b-3p), Hs_miR-20a_1 (hsa-miR-20a-5p), Hs_miR-21*_1 (hsa-miR-21–3p), Hs_miR-21_2 (hsa-miR-21–5p), Hs_miR-221_1 (hsa-miR-221–3p), Hs_miR-222_2 (hsa-miR-222–3p), Hs_miR-23a_2 (hsa-miR-23a-3p), Hs_miR-23b_2 (hsa-miR-23b-3p), Hs_miR-25_1 (hsa-miR-25–3p), Hs_miR-25_1 (hsa-miR-25–3p), Hs_miR-26a_2 (hsa-miR-26a-5p), Hs_miR-27a_1 (hsa-miR-27a-3p), Hs_miR-27a*_1 (hsa-miR-27a-5p), Hs_miR-27b_2 (hsa-miR-27b-3p), Hs_miR-30c_2 (hsa-miR-30c-5p), Hs_miR-31_1 (hsa-miR-31–5p), Hs_miR-320a_1 (hsa-miR-320a), Hs_miR-320_2 (hsa-miR-320b), Hs_miR-34a_1 (hsa-miR-34a-5p), Hs_miR-422b_1 (hsa-miR-378a-3p), Hs_miR-378c_1 (hsa-miR-378c), Hs_miR-378d_1 (hsa-miR-378d), Hs_miR-378f_1 (hsa-miR-378f), Hs_miR-423_1 (hsa-miR-423–3p), Hs_miR-452_4 (hsa-miR-452–5p), Hs_miR-629_2 (hsa-miR-629–5p), Hs_miR-7_2 (hsa-mir-7–5p), Hs_miR-92_1 (hsa-miR-92a-3p), Hs_miR-93_1 (hsa-miR-93–5p), Hs_miR-98_1 (hsa-miR-98), Hs_miR-99a_2 (hsa-miR-99a-5p), Hs_RNU6–2_1 (RNU6–2). All analyzed miRNAs are of human (*Homo sapiens*) origin and therefore the prefix “hsa” was omitted throughout the text.

### Small RNA deep sequencing and analysis

For each exosome sample, total RNA (containing the small RNA fraction) was extracted from one entire exosome preparation of 100 μl. Due to the low RNA yield, exosome samples were further concentrated to a volume of 6 μl using the RNeasy MinElute Cleanup Kit (Qiagen). The entire volume of resulting 6 μl concentrated exosomal total RNA (100 to 600 ng) was used as input for the library preparations. For cells, 1 μg total RNA in a volume of 6 μl was applied. Small RNA libraries were prepared using the NEBNext Multiplex Small RNA Library Prep Set for Illumina (NEB, Frankfurt/M., Germany) with custom multiplex adaptors and primers (NEBNext Multiplex Oligos for Illumina, Index Primers Set 1). Essentially, all materials not included in the set were purchased as recommended and the manufacturer’s guidelines were followed with a few modifications. Briefly, the Multiplex 3’ Adaptor was ligated to the RNA at 25°C for 1 h. After hybridization of the Multiplex RT Primer, the Multiplex 5’ Adaptor was ligated to the RNA. Afterwards, reverse transcription was performed using the SuperScript III Reverse Transcriptase (Life Technologies). The cDNA product was amplified by PCR using an optimized cycling protocol: initial denaturation for 3 min at 94°C, thirteen cycles of denaturation for 80 sec at 94°C, annealing for 30 sec at 62°C, extension for 15 sec at 70°C and final extension for 5 min at 70°C. Unique NEBNext Index Primers were applied for each of the six exosome samples (Index 1–6) and the four cellular samples (Index 1–4). Amplicons corresponding to adapter-ligated constructs from 21–30 nt RNA fragments were purified on a 6% TBE polyacrylamide gel (Life Technologies) and eluted at RT for 3 h. The gel slurry was passed through a 5 μm filter tube (IST Engineering, Milipitas, CA, USA) and precipitated overnight at -80°C. The size, DNA concentration and quality of each final small RNA library was determined twice using the High Sensitivity DNA Kit (Agilent) with the BioAnalyzer 2100. The concentration of each sample was adjusted to 10 nM and equal volumes of barcode-labeled samples were pooled for multiplexed sequencing in one lane. Sequencing (50 bp, single read) was performed on an Illumina HiSeq 2000 instrument (San Diego, CA). Raw sequencing reads were pre-processed and mapped using the function mapper.pl of the miRDeep2 package ([129], Max Delbrück Center, Berlin, Germany) as described by Weischenfeldt *et al*. [130]. Briefly, low quality reads were filtered out, the adaptor sequence was clipped and reads shorter than 18 nt were discarded. Accepted reads were mapped to known human miRNAs based on miRBase v.18.0 (www.mirbase.org/; [131–134]) using the function quantifier.pl in miRDeep2. Since one mismatch was allowed during the mapping procedure, the raw read file of each further analyzed miRNA was manually checked to assure that the reads truly annotated to the respective miRNA. The obtained raw read counts of each sample were normalized by dividing with the total number of reads mapping to known human microRNAs for each sample. Values are expressed as reads per million (RPM). Fold changes (FCs) were obtained by dividing the RPM of the si18E6/E7-treatment by the respective value of the siContr-1-treatment.

Genome mapping was performed on the pre-processed reads with miRDeep2 allowing two mismatches and up to five mapped positions in the genome. Read counts were obtained using python script rpkmforgenes.py (http://sandberg.cmb.ki.se/media/data/rnaseq/rpkmforgenes.py) applied to aligned data along with two major annotation sources: Gencode v.15 (http://www.gencodegenes.org/releases/15.html) for snoRNA; and RepeatMasker track (http://www.repeatmasker.org/) for scRNA, tRNA and snRNA.

### Statistical analyses

Statistical significance of the data was evaluated by the paired Student’s t-test using the Sigma Plot software (Systat Software Inc., San Jose, CA). P-values of p ≤ 0.05 (*), p ≤ 0.01 (**), and p ≤ 0.001 (***) were considered statistically significant.

## Supporting Information

S1 Dataset Raw read counts of each individual miRNA detected by small RNA deep sequencing. (XLSX)

S2 Dataset Overview of miRNAs reported to be HPV-dependent and/or deregulated in cervical cancer. (XLSX)

S1 Fig Comparison of intracellular and exosomal small RNA compositions of HeLa cells. (PDF)

S1 Table Library quality and mapping to the genome. (DOCX)

S2 Table Differentially affected cellular miRNAs upon silencing of endogenous *E6/E7* expression. (DOCX)

S3 Table miR-17~92 and miR-106b~25 levels upon silencing of endogenous *E6/E7* expression. (DOCX)

S4 Table Differentially affected exosomal miRNAs upon silencing of endogenous *E6/E7* expression. (DOCX)

S5 Table Studies on the global miRNA expression in cervical cancer tissues *in vivo* and/or in HPV-linked cell culture models *in vitro*. (DOCX)
