# Supplementary material for: Dependence of Intracellular and Exosomal microRNAs on Viral E6/E7 Oncogene Expression in HPV-positive Tumor Cells
Source: PLoS Pathog. 2015 Mar 11;11(3):e1004712. doi: 10.1371/journal.ppat.1004712 (PMC4356518; doi:10.1371/journal.ppat.1004712)
Supplement: S1 Fig — (PDF) [file ppat.1004712.s003.pdf]

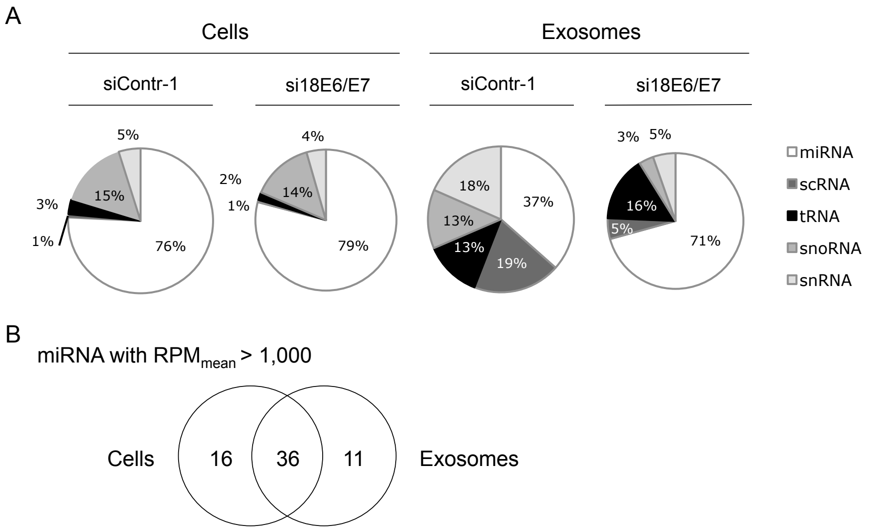

Figure S1. Comparison of intracellular and exosomal small RNA compositions of HeLa cells. (A) Changes in small RNA levels were determined by small RNA deep sequencing, 72 h post transfection of HeLa cells with si18E6/E7 or control siRNA siContr-1. Pie charts show percentages of reads mapping to miRNAs as well as other small cellular RNAs (scRNAs), transfer RNAs (tRNAs), small nucleolar RNAs (snoRNAs) and small nuclear RNAs (snRNAs). Small RNAs = 100 %. Data represent mean (n = 3 for exosomes; n = 2 for cells). **(B)** Venn diagram of cellular and exosomal miRNAs with > 1,000 RPM in each sample. Overlapping miRNAs (miRNAs with > 1,000 RPM in both cells and exosomes): let-7a-5p, let- 7b-5p, let-7g-5p, let-7i-5p, miR-100-5p, miR-101-3p, miR-103a-3p, miR-128, miR-143-3p, miR-151a-3p, miR-181a-5p, miR-181b-5p, miR-182-5p, miR-183-5p, miR-191-5p, miR-21- 5p, miR-22-3p, miR-221-3p, miR-23a-3p, miR-23b-3p, miR-24-3p, miR-27a-3p, miR-27a-5p, miR-27b-3p, miR-30a-5p, miR-31-5p, miR-320a, miR-320b, miR-378a-3p, miR-378c, miR-423-3p, miR-452-5p, miR-7-5p, miR-92a-3p, miR-99a-5p, miR-99b-5p (n = 36); miRNAs with > 1,000 RPM in cells only: miR-125a-5p, miR-125a-5p, miR-1307-3p, miR-140-3p, miR-17-5p, miR-186-5p, miR-193b-3p, miR-19b-3p, miR-21-5p, miR-25-3p, miR-29a-3p, miR-378d, miR-378f, miR-423-5p, miR-629-5p, miR-93-5p (n = 16); miRNAs with > 1,000 RPM in exosomes only: let-7d-5p, miR-10a-5p, miR-1246, miR-196a-5p, miR-20a-5p, miR-222-3p, miR-224-5p, miR-26a-5p, miR-30a-3p, miR-30c-5p, miR-98 (n = 11).
