# Supplementary material for: Dependence of Intracellular and Exosomal microRNAs on Viral E6/E7 Oncogene Expression in HPV-positive Tumor Cells
Source: PLoS Pathog. 2015 Mar 11;11(3):e1004712. doi: 10.1371/journal.ppat.1004712 (PMC4356518; doi:10.1371/journal.ppat.1004712)
Supplement: S1 Table — (DOCX) [file ppat.1004712.s004.docx]

**Table S1. Library quality and mapping to the genome.**

| Library | Sample | Biol. Repl. | Total reads | Reads after pre-processing |
| --- | --- | --- | --- | --- |
| 1 | **Exosomes**  **siContr-1** | I | 15,168,041 | 12,495,094 (82 %) |
| 2 |  | II | 43,844,625 | 33,424,143 (76 %) |
| 3 |  | III | 29,065,658 | 22,495,516 (77 %) |
| 4 | **Exosomes**  **si18E6/E7** | I | 24,333,222 | 18,735,296 (77 %) |
| 5 |  | II | 25,264,197 | 18,703,388 (74 %) |
| 6 |  | III | 22,791,005 | 17,388,436 (76 %) |
| 7 | **Cells**  **siContr-1** | I | 16,989,394 | 12,479,213 (73 %) |
| 8 |  | II | 36,471,533 | 25,139,400 (69 %) |
| 9 | **Cells**  **si18E6/E7** | I | 18,532,584 | 12,708,236 (69 %) |
| 10 |  | II | 25,817,875 | 17,264,822 (67 %) |
