# Supplementary material for: Dependence of Intracellular and Exosomal microRNAs on Viral E6/E7 Oncogene Expression in HPV-positive Tumor Cells
Source: PLoS Pathog. 2015 Mar 11;11(3):e1004712. doi: 10.1371/journal.ppat.1004712 (PMC4356518; doi:10.1371/journal.ppat.1004712)
Supplement: S2 Table — (DOCX) [file ppat.1004712.s005.docx]

**Table S2. Differentially affected cellular miRNAs upon silencing of endogenous *E6/E7* expression.**

| miRNA | Small RNA Deep Sequencing | | | | qRT-PCR | |
| --- | --- | --- | --- | --- | --- | --- |
|  | RPM_mean_^a^ | | FC_mean_^b^ | SEM | FC_mean_^b^ | SEM |
|  | siContr-1 | si18E6/E7 |  |  |  |  |
| **miR-7-5p** | **11660** | **2377** | **0.20** | **0.02** | **0.25** | **0.02** |
| **miR-629-5p** | **4160** | **1346** | **0.33** | **0.04** | **0.46** | **0.08** |
| **miR-378a-3p** | **30228** | **9841** | **0.34** | **0.07** | **0.46** | **0.01** |
| miR-378c | 11002 | 3654 | 0.35 | 0.07 | 0.45 | 0.11 |
| miR-378d | 3156 | 1232 | 0.40 | 0.07 | 0.57 | 0.15 |
| **miR-378f** | **2790** | **1109** | **0.41** | **0.08** | **0.53** | **0.04** |
| miR-1307-3p | 10188 | 4586 | 0.45 | 0.03 | 0.75 | 0.11 |
| miR-21-3p | 6833 | 3249 | 0.48 | 0.02 | 0.73 | 0.14 |
| **miR-17-5p** | **6945** | **3092** | **0.52** | **0.12** | **0.38** | **0.00** |
| miR-423-3p | 16765 | 9828 | 0.59 | 0.01 | 1.33 | 0.07 |
| miR-93-5p | 4188 | 2457 | 0.62 | 0.07 | 0.67 | 0.06 |
| miR-320b | 8811 | 5603 | 0.63 | 0.01 | 1.00 | 0.02 |
| **miR-186-5p** | **2850** | **1774** | **0.65** | **0.07** | **0.56** | **0.01** |
| miR-19b-3p | 2477 | 1496 | 0.68 | 0.18 | 0.61 | 0.21 |
| miR-27a-5p | 3282 | 2141 | 0.65 | 0.05 | 0.73 | 0.15 |
| **miR-23a-3p** | **4136** | **6061** | **1.57** | **0.24** | **1.52** | **0.00** |
| miR-125a-5p | 1839 | 2939 | 1.65 | 0.13 | 1.54 | 0.25 |
| **miR-23b-3p** | **3213** | **5154** | **1.71** | **0.27** | **1.72** | **0.01** |
| miR-27a-3p | 5923 | 9848 | 1.73 | 0.23 | 1.77 | 0.09 |
| **miR-27b-3p** | **6541** | **10927** | **1.73** | **0.21** | **1.50** | **0.01** |
| miR-31-5p | 3231 | 5816 | 1.96 | 0.38 | 2.26 | 0.42 |
| miR-221-3p | 2313 | 4327 | 2.00 | 0.33 | 1.30 | 0.10 |
| **miR-143-3p** | **4684** | **12025** | **2.70** | **0.47** | **2.48** | **0.26** |

Displayed are cellular miRNAs with > 1,000 RPM in each sample and > 1.5-fold up- or downregulation in small RNA deep sequencing upon *E6/E7* silencing. Indicated in bold are miRNAs in cells, which, in addition, showed a > 1.5-fold and significant deregulation in qRT-PCR. The latter are regarded as HPV *E6/E7*-dependent cellular miRNAs. Data represent mean ± SEM (n = 2 for small RNA deep sequencing, n = 3 for qRT-PCR).

^a^ Raw reads normalized to the total number of uniquely mapped reads per library, expressed as reads per million (RPM).

^b^ Fold changes (FCs) were obtained by dividing the values for the si18E6/E7-treatment by the respective siContr-1-treatment.
