# Supplementary material for: Dependence of Intracellular and Exosomal microRNAs on Viral E6/E7 Oncogene Expression in HPV-positive Tumor Cells
Source: PLoS Pathog. 2015 Mar 11;11(3):e1004712. doi: 10.1371/journal.ppat.1004712 (PMC4356518; doi:10.1371/journal.ppat.1004712)
Supplement: S3 Table — (DOCX) [file ppat.1004712.s006.docx]

Table S3. miR-17~92 and miR-106b~25 levels upon silencing of endogenous *E6/E7* expression.

| miRNA | | Small RNA Deep Sequencing | | | | qRT-PCR | |
| --- | --- | --- | --- | --- | --- | --- | --- |
|  |  | RPM_mean_^a^ | | FC_mean_^b^ | SEM | FC_mean_^b^ | SEM |
|  |  | siContr-1 | si18E6/E7 |  |  |  |  |
| miR-17~92 | **miR-17-5p** | **6945** | **3092** | **0.52** | **0.12** | **0.38** | **0.00** |
|  | **miR-20a-5p^c^** | **2645** | **1853** | **0.83** | **0.21** | **0.64** | **0.11** |
|  | miR-19b-3p | 2477 | 1496 | 0.68 | 0.18 | 0.61 | 0.21 |
|  | miR-92a-3p | 11071 | 7808 | 0.78 | 0.13 | 0.50 | 0.10 |
| miR-106b~25 | miR-25-3p | 3370 | 2232 | 0.69 | 0.07 | 0.64 | 0.18 |
|  | **miR-93-5p** | **4188** | **2457** | **0.62** | **0.07** | **0.67** | **0.06** |
|  | **miR-106b-5p** | **978** | **508** | **0.56** | **0.10** | **0.67** | **0.11** |

The listed miRNAs of the miR-17~92 and miR-106b~25 clusters were validated by qRT-PCR. miR-18a-5p and miR-19a-3p showed very low RPM values in small RNA deep sequencing and were not detected in qRT-PCR analyses. Indicated in bold are miR-17 family members, which share an identical seed region.

^a^ Raw reads normalized to the total number of uniquely mapped reads per library, expressed as reads per million (RPM).

^b^ Fold changes (FCs) were obtained by dividing the values for the si18E6/E7-treatment by the respective siContr-1-treatment.

Data represent mean ± SEM (n = 2 for small RNA deep sequencing, n = 3 for qRT-PCR).

^c^ miR-20a-5p was not included among the 52 most abundant cellular miRNAs (despite > 1,000 RPM_mean_) due to the fact that the RPM value in one replicate was < 1,000.
