# Supplementary material for: Dependence of Intracellular and Exosomal microRNAs on Viral E6/E7 Oncogene Expression in HPV-positive Tumor Cells
Source: PLoS Pathog. 2015 Mar 11;11(3):e1004712. doi: 10.1371/journal.ppat.1004712 (PMC4356518; doi:10.1371/journal.ppat.1004712)
Supplement: S4 Table — (DOCX) [file ppat.1004712.s007.docx]

**Table S4. Differentially affected exosomal miRNAs upon silencing of endogenous *E6/E7* expression.**

| miRNA | Small RNA Deep Sequencing | | | | qRT-PCR | |
| --- | --- | --- | --- | --- | --- | --- |
|  | RPM_mean_^a^ | | FC_mean_^b^ | SEM | FC_mean_^b^ | SEM |
|  | siContr-1 | si18E6/E7 |  |  |  |  |
| **miR-92a-3p** | **51114** | **12211** | **0.24** | **0.08** | **0.56** | **0.05** |
| **miR-7-5p** | **8509** | **2076** | **0.25** | **0.02** | **0.21** | **0.07** |
| miR-1246 | 13520 | 3491 | 0.28 | 0.09 | 1.52 | 0.19 |
| **let-7d-5p** | **7617** | **2487** | **0.33** | **0.06** | **0.57** | **0.01** |
| **miR-20a-5p** | **18352** | **7099** | **0.37** | **0.14** | **0.55** | **0.06** |
| miR-128 | 3542 | 1204 | 0.34 | 0.04 | 0.85 | 0.11 |
| miR-30c-5p | 9345 | 3476 | 0.37 | 0.07 | 1.29 | 0.04 |
| **miR-423-3p** | **7273** | **2713** | **0.37** | **0.02** | **0.22** | **0.05** |
| miR-191-5p | 8310 | 3351 | 0.40 | 0.09 | 0.97 | 0.08 |
| miR-103a-3p | 7289 | 3928 | 0.54 | 0.18 | 0.97 | 0.03 |
| miR-181b-5p | 5335 | 2918 | 0.53 | 0.09 | 1.32 | 0.04 |
| miR-222-3p | 5144 | 2723 | 0.53 | 0.07 | 1.00 | 0.15 |
| miR-98 | 3115 | 1711 | 0.54 | 0.11 | 0.91 | 0.11 |
| miR-196a-5p | 3831 | 2190 | 0.57 | 0.14 | 0.96 | 0.14 |
| miR-320a | 15484 | 8453 | 0.55 | 0.07 | 1.14 | 0.12 |
| let-7g-5p | 18783 | 10827 | 0.58 | 0.13 | 0.83 | 0.08 |
| miR-378c | 1919 | 1201 | 0.62 | 0.03 | n.d. | n.d. |
| miR-26a-5p | 9220 | 5969 | 0.65 | 0.11 | 1.03 | 0.01 |
| **miR-378a-3p** | **7293** | **4703** | **0.64** | **0.01** | **0.64** | **0.03** |
| let-7a-5p | 110479 | 72016 | 0.65 | 0.03 | 0.97 | 0.06 |
| let-7f-5p | 101146 | 65920 | 0.65 | 0.02 | 0.74 | 0.08 |
| miR-99a-5p | 28245 | 43914 | 1.55 | 0.11 | 1.56 | 0.15 |
| **miR-21-5p** | **343867** | **555023** | **1.61** | **0.12** | **1.58** | **0.00** |
| miR-100-5p | 30995 | 50515 | 1.62 | 0.11 | 1.49 | 0.08 |
| miR-143-3p | 3439 | 6729 | 2.08 | 0.34 | 0.69 | 0.02 |

Displayed are exosomal miRNAs with > 1,000 RPM in each sample and > 1.5-fold up- or downregulation in small RNA deep sequencing upon intracellular *E6/E7* silencing. Indicated in bold are miRNAs in exosomes, which, in addition, showed a > 1.5-fold and significant deregulation in qRT-PCR. The latter are regarded as HPV *E6/E7*-dependent exosomal miRNAs. Data represent mean ± SEM (n = 3).

^a^ Raw reads normalized to the total number of uniquely mapped reads per library, expressed as reads per million (RPM).

^b^ Fold changes (FCs) were obtained by dividing the values for the si18E6/E7-treatment by the respective siContr-1-treatment.
