# Supplementary material for: Dependence of Intracellular and Exosomal microRNAs on Viral E6/E7 Oncogene Expression in HPV-positive Tumor Cells
Source: PLoS Pathog. 2015 Mar 11;11(3):e1004712. doi: 10.1371/journal.ppat.1004712 (PMC4356518; doi:10.1371/journal.ppat.1004712)
Supplement: S5 Table — (DOCX) [file ppat.1004712.s008.docx]

**Table S5. Studies on the global miRNA expression in cervical cancer tissues *in vivo* and/or in HPV-linked cell culture models *in vitro.***

|  | Source | Year | First Author | Platform | Details |
| --- | --- | --- | --- | --- | --- |
| *In vitro* | [[22](#_ENREF_22)] | 2014 | Wang | miRNA microarray (LC Sciences),  Small RNA Deep Sequencing (Illumina) | HPV16- or HPV18-infected HVKs or HFKs in organotypic raft cultures |
|  | [[44](#_ENREF_44)] | 2013 | Yablonska | miRNA microarray (Agilent) | Ectopic expression of HPV16 E6, E7, or E6/E7 in HFK |
|  | [[39](#_ENREF_39)] | 2013 | Tang | TaqMan qRT-PCR Array  (Applied Biosystems) | Cervical cancer cell lines (n = 7) compared to normal cervical tissue (n = 2) |
|  | [[43](#_ENREF_43)] | 2013 | Gunasekharan | Small RNA Deep Sequencing (Solexa) | HPV31-transfected HFK in organotypic raft cultures |
|  | [[42](#_ENREF_42)] | 2011 | Dreher | miRNA microarray (Affymetrix) | HPV11, HPV16, and HPV45 transfected HaCaT keratinocytes |
|  | [[41](#_ENREF_41)] | 2008 | Martinez | miRNA microarray (Ambion) | HPV-positive cervical cell lines (n = 4) compared to normal cervical tissue and to HPV-negative C33A cervical cancer cells |
|  | [[40](#_ENREF_40)] | 2007 | Lui | Direct sequencing (Cloning) | Cervical cancer cell lines (n = 6) compared to normal cervical tissue (n = 5) |
| *in vivo* | [[24](#_ENREF_24)] | 2014 | Lin | miRNA microarray  (Beijing Boao Biotech) | Cervical cancer tissue (n = 3) compared to non-cancerous tissue from chronic cervicitis patients (n = 3) |
|  | [[38](#_ENREF_38)] | 2014 | Liang | miRNA microarray (Exiqon) | Cervical cancer tissue (SCC: n = 10) compared to normal cervical tissue (n = 10) |
|  | [[37](#_ENREF_37)] | 2014 | Park | miRNA microarray (Agilent) | Cervical cancer tissue (ADC: n = 4) compared to normal cervical tissue (n = 4) |
|  | [[28](#_ENREF_28)] | 2013 | Wilting | miRNA microarray (Agilent) | Cervical cancer tissue (SCC: n = 10, ADC: n = 9) compared to CIN II/III (n = 18) and normal cervical tissue (n = 10) |
|  | [[25](#_ENREF_25)] | 2012 | Ma | miRNA microarray (Agilent) | Cervical cancer tissue (SCC: n = 4) compared to normal cervical tissue (n = 4) |
|  | [[27](#_ENREF_27)] | 2012 | Lajer | miRNA microarray (Affymetrix) | Cervical SCC tissue (n = 10) compared to normal cervical tissue (n = 10) |
|  | [[29](#_ENREF_29)] | 2012 | Liu | miRNA microarray (Ambion) | Cervical cancer tissue (n = 15) compared to normal cervical tissue (n = 15) |
|  | [[31](#_ENREF_31)] | 2012 | Rao | miRNA microarray (CapitalBio) | Cervical cancer tissue (SCC: n = 11, ADC: n = 1, SA: n = 1) compared to normal cervical tissue (n = 13) |
|  | [[30](#_ENREF_30)] | 2011 | Li, Y | miRNA microarray (LC Sciences) | Cervical cancer tissue (SCC: n = 6) compared to CIN II/III (n = 6) and normal cervical tissue (n = 6) |
|  | [[32](#_ENREF_32)] | 2011 | Li, J-H | miRNA microarray (Illumina) | Cervical cancer tissue (SCC: n = 6) compared to normal cervical tissue (n = 6) |
|  | [[33](#_ENREF_33)] | 2010 | Pereira | miRNA microarray (National DNA-miRNA microarray Facility) | Cervical cancer tissue (SCC: n = 4) compared to CIN II/III (n = 5), CIN I (n = 9), and normal cervical tissue (n = 4) |
|  | [[34](#_ENREF_34)] | 2008 | Wang | miRNA microarray (LC Sciences) | Cervical cancer tissue (n = 4) compared to normal cervical tissue (n = 4) |
|  | [[35](#_ENREF_35)] | 2008 | Lee | TaqMan qRT-PCR Array  (Applied Biosystems) | Cervical cancer tissue (SCC: n = 10) compared to normal cervical tissue (n = 10) |
| *both* | [[23](#_ENREF_23)] | 2014 | Villegas-Ruiz | miRNA microarray (Affymetrix) | Cervical cancer tissue (n = 4), cervical cancer cell lines (n = 11) and HaCaT keratinocytes compared to normal cervical tissue (n = 4) |

HFK: human foreskin keratinocytes, HVK: human vaginal keratinocytes, CIN: Cervical intraepithelial neoplasia, SCC: squamous cell carcinoma, ADC: adenocarcinoma, SA: sarcoma
